# COVID-19 contagion across remote communities in tropical forests

**DOI:** 10.1038/s41598-022-25238-7

**Published:** 2022-12-01

**Authors:** Yoshito Takasaki, Christian Abizaid, Oliver T. Coomes

**Affiliations:** 1 Graduate School of Economics, University of Tokyo, Tokyo, Japan; 2 Department of Geography and Planning and School of the Environment, University of Toronto, Toronto, ON Canada; 3 Department of Geography, McGill University, Montreal, QC Canada

**Keywords:** Psychology and behaviour, Socioeconomic scenarios

## Abstract

Understanding COVID-19 contagion among poor populations is hampered by a paucity of data, and especially so in remote rural communities with limited access to transportation, communication, and health services. We report on the first study on COVID-19 contagion across rural communities without road access. We conducted telephone surveys with over 400 riverine communities in the Peruvian Amazon in the early phase of the pandemic. During the first wave (April–June, 2020), COVID-19 spread from cities to most communities through public and private river transportation according to their remoteness. The initial spread was delayed by transportation restrictions but at the same time was driven in unintended ways by government social assistance. During the second wave (August, 2020), although people’s self-protective behaviors (promoted through communication access) helped to suppress the contagion, people responded to transportation restrictions and social assistance in distinct ways, leading to greater contagion among Indigenous communities than mestizo communities. As such, the spatial contagion during the early phase of the pandemic in tropical forests was shaped by river transportation and social behaviors. These novel findings have important implications for research and policies on pandemics in rural areas.

## Introduction

Understanding COVID-19 contagion among poor populations in low- and middle-income countries and people’s reactions to the pandemic and corresponding government policies is critical to design effective policies to address post-pandemic problems and future pandemics^1–8^. In rural remote regions where people have limited access to transportation, communication, health services, and testing for COVID-19, available data are scant which hampers much-needed understanding^9,10^.

**Figure 1:**
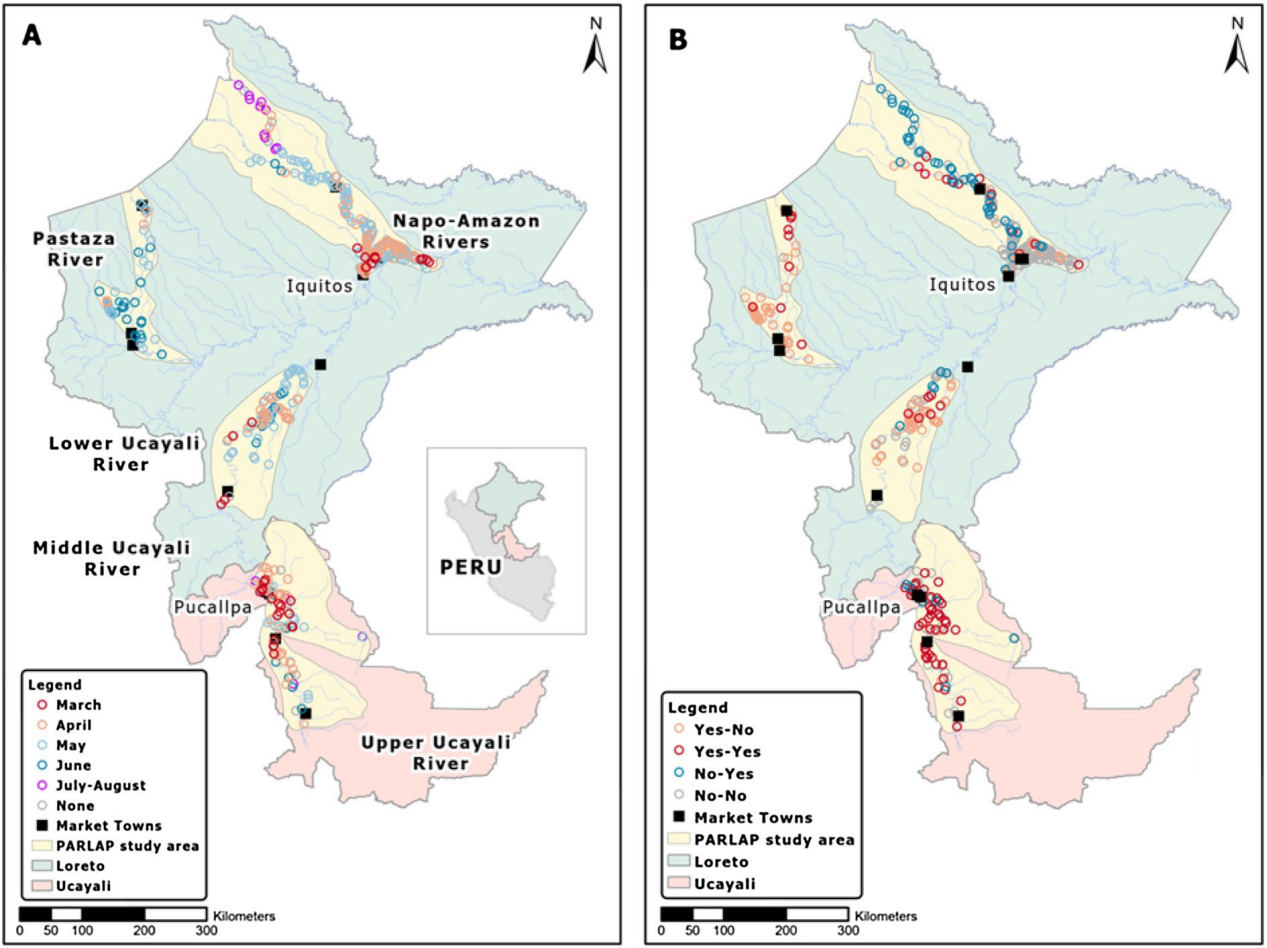
COVID-19 spread and evolution across communities. First COVID-19 case in 2020 (**A**) and COVID-19 evolution between the baseline survey (in July 2020) and the follow-up survey (in August 2020) (for example, Yes–No means any case at the baseline and no case at the follow-up) (**B**). In A, June includes two communities which experienced first case in July; July–August means first case which occurred between the baseline and follow-up surveys.

Our study examined the spread and evolution of COVID-19 during the early phase of the pandemic across rural communities without road access in the Peruvian Amazon. In this remote environment where rivers serve as roads, transportation restrictions could potentially prevent the spread of the virus by effectively isolating communities. Immediately after a national lockdown was lifted, we conducted telephone surveys with over 400 communities in four major river basins—the Amazon, Napo, Pastaza and Ucayali (near 120,000 km^2^, or about 2.3 times the area of Costa Rica) (Fig. 1; ‘Study area’ and ‘Surveys’ in Methods)^11^. Peru ranks among the countries most severely affected by COVID-19, despite instituting one of the earliest and longest lockdowns in Latin America^12–15^. In Peru, riverine populations in Amazonia which rely on wild resources for their livelihoods are among the poorest and most neglected (‘Study area’ in Methods)^16^.

Our study has three related objectives. First, we assess the spatial contagion of COVID-19 across rural communities relying solely on river transportation. Extant works on the spatial spread of disease highlight the role of human movement through air and ground transportation, especially across and within urban areas, suggesting policies for mitigation targeted towards highly connected areas^17–24^. Research and policy pay lesser attention to isolated areas, such as those we study, and are constrained by paucity of data on human and pathogen movements^25^. To address this challenge, our large-scale community surveys capture contagion across communities over large geographical areas (‘Study area’ and ‘Surveys’ in Methods).

Second, we examine both government policies restricting human movement (transportation restrictions, in particular) and local people’s self-protective behaviors (e.g., social distancing) which are known to be critical in restricting person-to-person interactions to suppress contagion^26–29^. We add to the literature by showing how these two factors shaped the spatial contagion of COVID-19 across rural communities through public and private river transportation in interrelated ways.

Lastly, we compare Indigenous and mestizo communities (‘Study area’ in Methods). Mestizos (folk peoples; locally known as *ribereños*) are descendants over many generations of Iberian and Indigenous peoples living in the region^30^. Concerns about the fate of Indigenous peoples, especially in Amazonia, are warranted and have been prominent in media reports and research on COVID-19, especially during early stages of the pandemic, but tend to overlook the larger mestizo population^31–34^. Our large-scale community surveys allow us to make comparisons between these two populations across communities, which is uncommon in the broad literature on Amazonia. We find that transportation restrictions and self-protective behaviors shaped the spatial contagion of COVID-19 in distinct ways for these two populations.

## Results

### COVID-19 spread

Since the first COVID-19 case was reported in the Peruvian Amazon on March 16, 2020, the virus spread in two waves in 2020 (April-June; August; Supplementary Fig. 1). Mortality was highest early during the first wave, especially in the cities of Iquitos and Pucallpa. A national lockdown was declared on March 16, 2020, lasting until early May, when restrictions were gradually relaxed^15^. At the end of June 2020, the lockdown was lifted, though various regional restrictions such as curfews were maintained. We conducted two rounds of community telephone surveys: a baseline survey in July 2020 (between the two waves) and a follow-up survey in August 2020 (during the second wave) (‘Surveys’ in Methods). Our analysis sample is a balanced panel of 435 communities (240 Indigenous, 195 mestizo) (Fig. 1). Compared to mestizo communities, Indigenous communities are found in more remote areas in all river basins, especially in the Napo, Pastaza, and Upper Ucayali (Supplementary Fig. 2).

**Figure 2:**
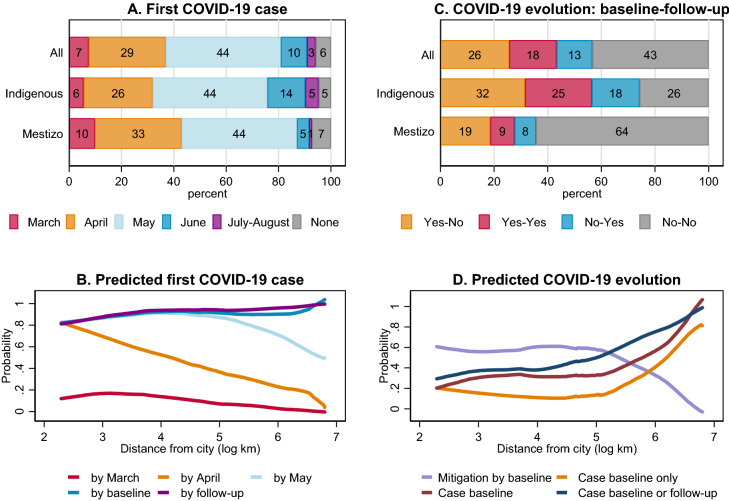
COVID-19 spread and evolution. First COVID-19 case in 2020 by indigeneity (**A**), nonparametric relationship of first case with distance from city (**B**), COVID-19 evolution between the baseline survey (in July 2020) and the follow-up survey (in August 2020) by indigeneity (for example, Yes–No means any case at the baseline and no case at the follow-up) (**C**), and nonparametric relationship of COVID-19 evolution with distance from city (**D**). In A June includes two communities which experienced first case in July and July–August means first case occurred between the baseline and follow-up surveys. In B and D Lowess (locally weighted scatterplot smoothing) smoothers are shown; one community with log distance from city smaller than 2 km is dropped for exposition.

We focus on any incidence of COVID-19 case in the community, including suspected ones (see Supplementary Note 1 for mortality). With limited health facilities and testing for COVID-19 in the study area (19% of communities had health facilities, generally understaffed and with limited supplies to provide adequate health services), information about confirmed cases is incomplete and data on the number of cases can be inaccurate. Although suspected case incidence may be also inaccurate, any case captures people’s perceived risk of infection in the community that would have underlain their behaviors. The first COVID-19 case had occurred in most communities (91%) by June, i.e., within three months, during the first wave (Fig. 2A). We estimate the predictors of initial spread across communities using regression analyses (‘Empirical design’ in Methods). Multivariate regression analysis better captures the relationship between outcome variable (initial spread) and each predictor (e.g., distance) by controlling for other factors that may be correlated with both. Our sensitivity analysis indicates that most regression estimates are robust to omitted variable bias (see Supplementary Note 2).

**Figure 3:**
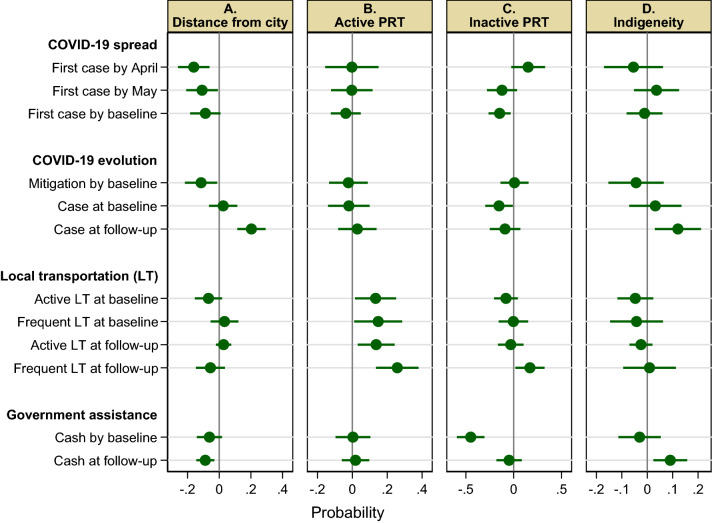
Remoteness, transportation, and indigeneity. The estimated impacts of distance from city (log km) (**A**), active public river transportation (PRT) (0/1) (**B**), inactive PRT (0/1) (**C**), and indigeneity (0/1) (**D**) on COVID-19 spread and evolution, local private river transportation (LT), and government cash assistance (0/1), with 95% confidence intervals based on robust standard errors. Full regression results are reported in Supplementary Tables 1 and 3.

Without road access in the study area, local residents rely solely on river transportation for travelling. The river network distance from cities (Iquitos or Pucallpa) varies markedly by community (Supplementary Fig. 3A; range: 3–898 km, mean: 261 km). The timing of initial spread across communities depended on their remoteness: the nonparametrically predicted probability of spread by April and May shows negative steep distance gradients (Fig. 2B). The regression estimates for the initial spread by April, May, and the baseline survey are consistent with these bivariate nonparametric relationships: a 100% increase in the distance from cities decreased the probability of spread by April, for example, by 0.16 (or 44% of the mean of the dependent variable); in contrast, distances from nearest market town, district capital, and nearest community were not significant predictors (Fig. 3A, Supplementary Fig. 4, Supplementary Table 1). Dendritic river network structure (captured by river order), trade network (with city markets), and the availability of health facility were not significant predictors either (Supplementary Table 1). These results indicate that the primary factor determining the speed of spread was distance from the source of the virus, i.e., cities. The spatial distribution of the timing of spread in each basin is consistent (Fig. 1A).

As Indigenous communities tend to be located further from cities than mestizo communities (Supplementary Figs. 2, 5; mean: 353 km vs.147 km), it took longer for the virus to reach Indigenous communities (until May), but by the time of the baseline survey in July the difference between these two types of communities had vanished (Fig. 2A). Our regression results show that for a given distance from cities, there was no significant difference between Indigenous and mestizo communities (Fig. 3D), indicating that their difference in the timing of spread was due to their difference in remoteness, not indigeneity per se. This illustrates the importance of multivariate regression analysis relative to bivariate correlation analysis, which would suggest significant relationships between the timing of spread and indigeneity.

A variety of watercraft—both private and public boats—navigate Amazonian rivers^35,36^ (‘River transportation’ in Methods). Public river transportation is effectively the only way for local people to travel from remote communities to cities and was available for 80% of all communities (Indigenous and mestizo) before the pandemic (Supplementary Fig. 6A). The government imposed mobility restrictions to suppress COVID-19 contagion. During the strict lockdown period, public river transportation virtually stopped. At the time of the baseline survey after the lockdown was lifted, public river transportation had been partially reactivated: the restriction was relaxed in 64% of communities and it was maintained in 16% (Supplementary Fig. 6A; see Supplementary Notes 3 and 4 for spatial distribution and predictors, respectively).

Although the timing of initial spread in communities with active public transportation was similar to that in communities without access to public transportation before the pandemic, in communities with inactive public transportation under the maintained restriction, the spread was delayed; this was especially so in May and June, when mobility restrictions were gradually relaxed (Fig. 3B,C; by April, spread had become quite common in communities with inactive public transportation, so transportation restrictions were maintained in these communities). The delaying effect of transportation restrictions was large: to decrease the probability of initial spread by the time of the baseline survey, for example, by 0.15, the distance from cities needed to be greater by 170 km (-0.15/-0.087 × 100; 65% of mean distance; Supplementary Table 1). As such, transportation restrictions were effective in delaying initial spread.

### COVID-19 evolution

Although 91% of communities had experienced COVID-19 by the time of the baseline survey, a case was prevalent among 44% at that time; that is, it had been mitigated among the remaining 47% (Fig. 2A,C). Whereas mitigation was not related to the timing of initial spread (Supplementary Note 5), it was more common in communities closer to cities, especially near Iquitos (Figs. 1,2), and in mestizo communities than Indigenous communities (64% vs. 33%; Fig. 2A,C, S2). For a given distance from cities, there was no significant difference between these two types of communities (Fig. 3D), indicating that their difference in mitigation was due to their difference in remoteness, not indigeneity, as found for initial spread.

At the time of the follow-up survey, COVID-19 was mitigated in 26% of communities but at the same time the virus had spread in 13% (initial spread 3% and resurgence 10%); it was persistent and not prevalent in 18% and 43%, respectively, over time (Fig. 2C). Mitigation during this period was more common in communities far from cities (Fig. 2D); thus, the location of mitigation shifted to remote areas (Fig. 1B; Supplementary Note 3).

Mitigation, persistence, and spread were more common in Indigenous communities than mestizo communities (Fig. 2C). Distinct from initial spread, significant differences emerged across basins: whereas mitigation was common in the Pastaza and Lower Ucayali, the virus persisted in the Middle and Upper Ucayali and spread in the Napo (Supplementary Figs. 2B, 2D). At the time of the follow-up survey, COVID-19 was more prevalent in communities far from cities and Indigenous communities, for a given distance from cities (Fig. 3A,D, Supplementary Table 1). Hence, distinct from initial spread during the first wave and mitigation by the time of the baseline survey, a significant difference by indigeneity emerged during the second wave.

### Social behaviors and transportation restrictions

People in communities employed a wide range of self-protective behaviors. We construct two indices (z-score): (1) preventive measures mostly based on individual behaviors (e.g., handwashing) before the baseline survey (mid-March-July), which captures initial adoption, and at the time of the follow-up survey, and (2) social restrictions consisting of community-level restrictions (e.g., no communal meeting) at the time of each survey (‘Social behaviors’ in Methods). Over time, preventive measures became weaker and social restrictions became somewhat stronger (Supplementary Fig. 7A; see Supplementary Notes 3 and 6 for spatial distribution and individual self-preventive behavioral measures, respectively). Both preventive measures and social restrictions became relatively stronger in Indigenous communities than in mestizo communities (Supplementary Figs. 7B,C; Supplementary Note 6).

**Figure 4:**
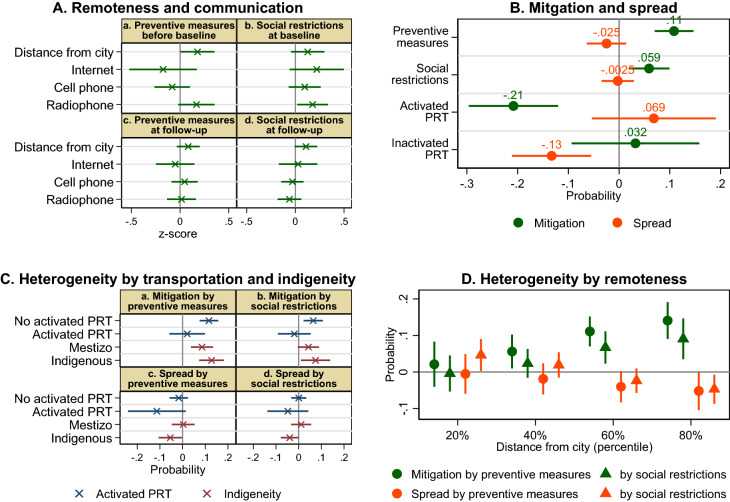
Social behaviors and transportation restrictions. The estimated predictors of self-protective behaviors (z-score) (**A**), the estimated impacts of a change in self-protective behaviors (z-score) and public river transportation (PRT) (0/1) on COVID-19 mitigation and spread between the baseline and follow-up surveys (0/1) (**B**), heterogenous impacts by PRT and indigeneity (**C**), and heterogenous impacts by distance from city (percentile) (**D**), with 95% confidence intervals based on robust standard errors.

Almost three quarters of communities had communication access (internet, cell phone, or radiophone) (the surveys covered communities without communication access as discussed in ‘Surveys’ in Methods; see Supplementary Note 3 for spatial distribution). Radiophones are typically available in some communities beyond the reach of cell phone coverage. When we consider these three modes separately, preventive measures at the baseline were stronger in communities with radiophone, but not internet or cell phone access, although social restrictions were stronger in communities with any of these three modes (Fig. 4A). In contrast, preventive measures and social restrictions at the follow-up were unrelated to these three communication modes. They were not related to the presence of a health facility over time (Supplementary Table 2), whereas both were strong in communities far from cities over time (Fig. 4A).

The status of public river transportation changed between the baseline and follow-up surveys: it was inactivated (the restriction which had been relaxed before the baseline was reimplemented) and activated (the restriction which had been maintained at the baseline was relaxed) among 10% and 13% of communities, respectively, and remained active and inactive among 54% and 3%, respectively (Supplementary Fig. 6A). These patterns were similar between Indigenous and mestizo communities (see Supplementary Notes 3 and 4 for spatial distribution and predictors, respectively).

We examine the impacts of a change in preventive measures and social restrictions (indices), and activated and inactivated public river transportation (indicator variables) on the mitigation and spread (mainly resurgence) of COVID-19 between the baseline and follow-up surveys (‘Empirical design’ in Methods). This first difference specification addresses the endogeneity of social behaviors by controlling for time-invariant community heterogeneity including unobserved factors that could cause omitted variable bias.

Inactivated public transportation reduced the probability of spread (by 0.13, or almost 100% of the mean), but did not increase mitigation (Fig. 4B). Thus, reimplementing the transportation restriction was effective to prevent (eliminate) the spread. Consistently, COVID-19 was less prevalent in communities with inactive public transportation at the baseline (Fig. 3C). In contrast, activated public transportation decreased the probability of mitigation (by 0.21, or over 90% of the mean). Thus, lifting transportation restrictions almost entirely counteracted mitigation.

Both preventive measures and social restrictions increased mitigation but did not reduce spread (Fig. 4B; see Supplementary Note 7 for results for individual self-preventive behavioral measures). These impacts are comparatively smaller than those of transportation restrictions: to entirely counteract the diminishing effect of activated public transportation on mitigation, preventive measures and social restrictions, respectively, need to be increased by about 2 and 3.5 standard deviations (0.21/0.11 and 0.21/0.06). Thus, self-protective behaviors were effective to mitigate contagion, but not to prevent it.

## Discussion

### River transportation

The lack of significant difference in the speed of initial spread between communities with active public river transportation and those without access to public transportation indicates that the virus was brought to the former communities from cities by public transportation and quickly spread to the latter communities. This was possible because active public transportation connecting to market towns (including Iquitos and Pucallpa; Fig. 1) attracted people from nearby communities. Indeed, local private river transportation (small personal boats; ‘River transportation’ in Methods) was more common in communities with active public transportation, but not in those with inactive public transportation at both baseline and follow-up surveys (Fig. 3B,C, Supplementary Table 3). Anecdotal evidence suggests that near cities personal boats were still used during the lockdown period (incomplete compliance). These results indicate that COVID-19 contagion from cities was driven by market access and the reduction of market access through transportation restrictions effectively delayed the initial spread of COVID-19 and shaped its evolution afterwards. This is buttressed by analyzing different public river transportation modes separately (Supplementary Note 8). The potential spread of COVID-19 through river transportation and among small-scale fishermen at landing sites has been reported in Brazil^37^ and in Africa^38^, respectively.

Government cash assistance served as an important safety net for poor households during the pandemic^39–41^. People had to travel to the city or a district capital to collect cash support at a financial institution. People had done so in 82% of communities before the baseline survey. Cash assistance was much less common (by 45%) in communities with inactive public transportation, as found for initial spread, but was not related to distance from cities or district capital or to indigeneity (Fig. 3B,C, Supplementary Table 3). These results indicate that the virus spread unintentionally by people traveling to collect cash support, as has been the case among Indigenous peoples in other part of the Peruvian Amazon^42^. Ironically, transportation restrictions were effective at reducing the spread by constraining people’s access to social assistance. This finding is buttressed by the results of different public river transportation modes (see Supplementary Note 8; see Supplementary Note 9 for other forms of social assistance and return migration).

At the time of the follow-up survey, people had recently received cash assistance in 12% of communities (during the previous 7 days; in most of these communities, people had received cash assistance also before the baseline survey). Cash assistance was more common (by 9%) in Indigenous communities than mestizo communities; it was also more common in communities closer to cities, but was not related to public river transportation (Fig. 3, Supplementary Table 3). These results suggest that continued reliance on cash assistance among Indigenous people during the second wave led to the higher prevalence of COVID-19 among them.

### Social behaviors and transportation restrictions

Poor communication infrastructure is likely to have caused important delays and gaps in information, or misunderstandings about COVID-19^43,44^. Distinct from internet and cell phone, information was received by community leaders through radiophone. The effectiveness of radiophone for promoting preventive measures (Supplementary Fig. 7D) suggests that community leaders with an information advantage could better coordinate people’s adoption of individual behaviors which they were not familiar with (e.g., wearing a mask). Such information advantage may not have been significant for restricting community-level social behaviors which were common before the pandemic (e.g., communal work).

The impacts of preventive measures (social restrictions) on the mitigation of contagion were stronger with greater social restrictions (preventive measures), indicating that these two sets of self-protective individual and community behaviors were complementary to each other (Supplementary Fig. 8A). The effectiveness of self-protective behaviors was differentiated according to whether public river transportation was activated, but not to whether it was inactivated, as follows. On one hand, preventive measures and social restrictions increased mitigation only in communities with no activated transportation (Fig. 4C), indicating that lifting transportation restrictions nullified their mitigating effects; put differently, transportation restrictions were complementary to self-protective behaviors for mitigating contagion. At the same time, activated transportation reduced mitigation regardless of the level of adoption of self-protective behaviors (Supplementary Fig. 8B). On the other hand, preventive measures reduced spread only in communities with activated transportation (Fig. 4C). Whereas inactivated transportation (reimplementing transportation restriction) reduced the spread regardless of the level of adoption of self-protective behaviors, activated transportation increased the spread only when preventive measures and social restrictions were weak (Supplementary Fig. 8C). Thus, self-protective behaviors were complementary to transportation restrictions in preventing the spread.

We conjecture that the impacts of transportation restrictions on contagion were stronger in remote communities because people there rely more on public river transportation for market access. Indeed, both the diminishing effects of activated public transportation on mitigation and those of inactivated transportation on spread were stronger in communities far from cities (Supplementary Fig. 9A). Moreover, in remote communities, activated public transportation increased spread and inactivated transportation increased mitigation. We also conjecture that self-protective behaviors were effective to suppress contagion in remote communities because preventive measures and social restrictions were stronger there (Fig. 4A). Indeed, both sets of behaviors increased mitigation in a greater magnitude in communities further from cities (Fig. 4D); moreover, they reduced spread only in remote communities.

Although the effectiveness of preventive measures and social restrictions for mitigation was not differentiated by indigeneity, both were effective at reducing the spread in Indigenous communities only (Fig. 4C). This is consistent with the observation that they became relatively stronger in Indigenous communities over time, and that they were more effective in remote communities. Despite such differences in self-protective behaviors, COVID-19 became more prevalent in Indigenous communities at the follow-up. This inconsistency is explained by transportation restrictions’ impact being greater than of self-protective behaviors: activated public transportation increased spread only in Indigenous communities, especially in remote places (Supplementary Fig. 9B,C), as found in the upper Napo (Supplementary Figs. 2B, 10A). Inactivated transportation rather reduced mitigation in Indigenous communities close to cities (Supplementary Fig. 9D). It is possible that Indigenous peoples responded to limited market access in a distinct manner than mestizos, which facilitated contagion during the second wave, as found for their responses to social assistance.

### Limitations

Three limitations in our study are to be noted. First, as in any survey, we cannot rule out the possibility of measurement errors, though community telephone surveys such as those we conducted better circumvent reporting bias (e.g., social desirability bias) than standard household telephone surveys (‘Surveys’ in Methods). Second, our data do not allow us to examine the extent to which the different patterns found between Indigenous and mestizo communities may be underlain by contrasting cultural and social norms. Our community-level survey data also preclude us from exploring household-/individual-level factors. Finally, although our analysis sample captures well the dynamics of spatial contagion, it is not necessarily regionally representative (‘Surveys’ in Methods). Concern about external validity has been a common issue with telephone surveys during the pandemic.

### Implications

Our study on rural communities without road access in the Peruvian Amazon revealed that COVID-19 contagion during the early phase of the pandemic was shaped by river transportation and social behaviors. Our findings have the following implications for research and policy related to pandemics more broadly.

1. Community-level analysis on case incidence is effective for capturing not only the spatial spread of disease, but also its spatial evolution (mitigation, persistence, resurgence) and people’s self-protective behaviors. This approach is practical for rural research during pandemics when researchers must rely on telephone surveys to collect data. This approach can be applied also to understand rural people’s vaccination behaviors^45–47^.
2. The virus can spread from cities to rural communities through limited river transportation driven by people’s actions for livelihood and coping. Remoteness delays the speed of contagion but does not prevent the spread to rural communities altogether.
3. It is possible to significantly delay and suppress contagion across rural communities by restricting their transportation access to cities. Failing to employ such restrictions across cities led to the rapid spread of COVID-19 in Brazil^21^. Balancing this health gain against associated economic costs of limited market access and various costs of reduced social connection (e.g., social cohesion, mental health)^2^ is a central tradeoff in policymaking in remote rural areas under pandemics.
4. Rural people’s self-protective behaviors are also key in suppressing contagion. Although improving communication access can promote such behaviors and facilitate data collection^43^, who receives information and how it is used may shape the adoption of unfamiliar individual behaviors^44^. This applies also to communication for promoting COVID-19 vaccination^48,49^.
5. The effectiveness of self-protective behaviors will likely depend on the combination of individual and community behaviors, and government policies such as transportation restrictions. Policy makers need to design pandemic policies considering their potential complementarities and possible unintended side effects. Developing a digital payment platform in places where it does not yet exist is essential for the effective and timely distribution of cash assistance while avoiding negative public health externalities^50,51^.
6. In pandemics, people in different socio-cultural groups may adopt self-protective behaviors including vaccination and respond to government policies in distinct ways. In Peru, increased vulnerability of Indigenous people is a major concern as found also in Brazil^52^.

## Methods

### Ethical approval

This study was approved by the Research Ethics Board of McGill University (#290–114) and performed in accordance with relevant guidelines and regulations. We obtained informed consent from all participants.

### Study area

The Departments of Loreto and Ucayali cover about 85% of the area of the Peruvian Amazon (Fig. 1) consisting of humid tropical forest and extensive wetlands at < 200 m of elevation. Iquitos (population: 437,400) in Loreto and Pucallpa (population: 211,700) in Ucayali serve as major markets and administrative centers^53^. Iquitos can be reached only by riverboat or by air; Pucallpa has also been connected with Lima, the capital city of Peru, by road since the 1940s. Small towns (5000–30,000 inhabitants) which function as district capitals and secondary markets and many smaller communities (100–300 inhabitants) with limited access to public and health services line the main rivers and tributaries. Forest peoples (both Indigenous and mestizo) practice agriculture, fishing, hunting, timber and non-timber forest product gathering, and small livestock raising for subsistence and cash earnings, sending produce to market by boat^16,54,55^.

### Surveys

#### PARLAP

Our COVID-19 surveys were part of the Peruvian Amazon Rural Livelihoods and Poverty (PARLAP) project (https://parlap.geog.mcgill.ca)^56^. We selected four major river basins—the Amazon, Napo, Pastaza, and Ucayali—to capture the diversity of ecological conditions, economic activities, history, and ethnicity of its peoples (Fig. 1). We sought to cover all communities in the study area. In each river basin, field teams were guided by data from the 2007 population census from the Peruvian *Instituto Nacional de Estadística e Informática* (INEI)^53^, maps from the *Instituto del Bien Común* (IBC) for their census of Indigenous communities^57,58^, and Google Earth imagery, supplemented by local enquiries by the teams to identify unmapped settlements. The community survey conducted from December 2012 through March 2014 reached a total of 919 communities (436 Indigenous, 470 mestizo, and 13 colonist), which we estimate represents 92% of all communities in the study area (i.e., a near census). Each community was geo-referenced using a handheld Garmin GPS unit. The survey collected information through a focus group-based in-person interview among community leaders and elders following a structured questionnaire. We use data from this community survey to construct some variables.

#### COVID-19 surveys

Excluding district capitals and communities with a health center from 919 communities covered in the community survey, the remaining 893 communities were eligible for the COVID-19 survey^11^. Our baseline telephone survey, which was conducted in July 2020, covered 469 communities (53% of the target communities; 369 in Loreto, 100 in Ucayali). We subsequently conducted a follow-up telephone survey in August and early September 2020 that reached 435 of the 469 communities in the baseline sample (7% attrition). The analysis sample is the panel sample of 435 communities.

Our surveys sought information from community leaders following a structured questionnaire. With the suspension of public telephone service since November 2019 and an unreliable radiophone system, we relied mostly on cell phone contact. Our field teams visited ports and markets in Iquitos and Pucallpa to find people from the target communities. Some telephone interviews were arranged through an intermediary when people from the target communities visited a town where the intermediary lived. In these ways the surveys also contacted people in communities with no telephone access.

#### Sample representativeness

The non-randomly sampled communities in the COVID-19 survey are not representative of the PARLAP study area: communities in the Napo basin (the Middle and Upper Ucayali basins) and Indigenous communities are more (less) likely to have been sampled in the baseline survey and be found in the follow-up survey (Supplementary Table 4). The panel sample was not correlated with distance from cities, access to public river transportation, telephone access, and the availability of health facility. These results suggest that the findings from the COVID-19 sample may not be generalizable to the whole PARLAP study area. The limitation of external validity is a common problem of telephone surveys during the pandemic.

#### COVID-19 surveys in low- and middle-income countries

Most surveys in other extant and on-going research projects on COVID-19’s socio-economic impacts in low- and middle-income countries listed at Innovations for Poverty Action (https://www.poverty-action.org/recovr/research-projects) have been conducted at the household (or firm) level and a small number of projects have included community questionnaires^59^. Our COVID-19 survey is the first to our knowledge to employ a large-scale community telephone survey.

### River transportation

In the region, public river transportation consists of three modes: large river boats (*lanchas*), small river boats (*colectivos*), and speed boats (*rápidos*)^36^. Large river boats offer regular passenger and cargo service between cities and towns, stopping in selected towns and communities along main rivers, many of which function as intermediary hubs between the cities and other rural communities. Small river boats are more common within a day’s travel from cities and towns for both passengers and cargo; serving as river buses, they run along both main rivers and tributaries which are not accessible by large river boats. In the last decade, powerful speed boats have begun offering regular passenger service along main rivers between cities and towns within a 10-h trip. Travel is about 3–4 times faster and more comfortable than on a large or small river boat but the cost is prohibitive for most rural people. Local private river transportation consists of small personal boats powered by a small air-cooled engine attached to a long shaft and a small propeller (*peque-peques*).

### Social behaviors

People in rural communities employed preventive measures and restricted social activities. We construct two indices based on the pooled baseline and follow-up data. A preventive measure index is a z-score constructed by taking the first principal component of 8 indicator measures: hand washing, use of a mask, and social distancing measures—avoiding physical greetings, maintaining enough distance, staying at home, avoiding gatherings, avoiding travel, and restricting entry to the community—before the baseline survey (mid-March-July) and at the time of the follow-up survey. Our social restriction index is a z-score constructed by taking the first principal component of 7 indicator measures: primary and secondary school closure at the time of the surveys (which takes 1 if there was no school), not playing soccer and volleyball during the previous 7 days, and no gatherings for communal work, community meetings, and church services during the previous 7 days (which takes 1 if there was no church).

### Empirical design

We estimate predictors of outcomes using Ordinary Least Squares (OLS) regression specifications of the form

1 $${Y}_{ict}=\mathrm{\alpha }+\upbeta \cdot {X}_{it}+{\phi }_{c}+{\varepsilon }_{ic}$$

where *Y*_*ict*_ is outcome variables, such as an indicator variable for case incidence and self-protective index (z-score), of community *i* in basin *c* at period *t*; *X*_*it*_ is a vector of predictors, including interviewer fixed effects which capture interview heterogeneity including potential reporting bias (8 interviewers); *ϕ*_*c*_ is a vector of basin fixed effects which capture basin heterogeneity (6 basins); and *ε*_*ic*_ is an error term. Inference is based on robust standard errors. Supplementary Fig. 6A shows the distribution of transportation variables used as predictors and Supplementary Table 5 shows the definition and descriptive statistics of other predictors (the construction of transportation variables and some of other predictors is provided below). Full regression results are reported in Supplementary Tables 1, 2, 3, 6, and 7. The number of observations for some outcome variables is slightly smaller than 435 due to missing values.

We estimate impacts of social behaviors and transportation restrictions on COVID-19 case using first difference specifications of the form

2 $${Y}_{ict}{-Y}_{ict-1}=\mathrm{\alpha }+\upbeta \cdot \left({Z}_{ict}{-Z}_{ict-1}\right)+\upgamma \cdot \left({X}_{ict}{-X}_{ict-1}\right)+\left({\varepsilon }_{ict}{-\varepsilon }_{ict-1}\right)$$

where *Y*_*ict*_ is case incidence of community *i* in basin *c* at period *t* (*t*: follow-up, *t* − 1: baseline); *Z*_*it*_ is a vector of self-protective behavior indices—preventive measures and social restrictions (z-score); *X*_*it*_ is a vector of public river transportation; and *ε*_*ict*_ is an error term. Controlling for time-invariant community heterogeneity—both observed and unobserved factors—this first difference specification allows us to capture the relationships of COVID-19 case with social behaviors and transportation restrictions within communities. The first difference of binary outcomes takes −1, 0, or 1. We consider two binary measures: (1) an indicator variable which takes 1 if the difference takes −1, that is, the original measure decreases from 1 to 0 (i.e., mitigation); and (2) an indicator variable which takes 1 if the difference takes 1, that is, the original measure increases from 0 to 1 (i.e., spread). Analogously, we consider two binary measures for public river transportation: (1) an indicator variable which takes 1 if the original measure decreases from 1 to 0 (i.e., inactivated); and (2) an indicator variable which takes 1 if the original measure increases from 0 to 1 (i.e., activated). Inference is based on robust standard errors. The number of observations is 367 communities with non-missing values in self-protective behavior indices at the baseline and follow-up.

When we examine complementarity of preventive measures and social restrictions, we add their interaction term to Eq. (2), estimating the marginal effects of the preventive measure (social restriction) index according to the value of the social restriction (preventive measure) index. When we examine complementarity of social behaviors and public transportation, we add an interaction term of one of the self-protective behavior indices and activated/inactivated transportation to Eq. (2), estimating the marginal effects of the self-protective behavior index (transportation variable) according to the value of the transportation variable (self-protective behavior index). When we conduct heterogeneity analysis by one of community-level time-invariant factors such as indigeneity, we add an interaction term of one of the self-protective behavior indices/transportation variables and the time-invariant factor to Eq. (2), estimating the marginal effects of the self-protective behavior index/transportation variable according to the value of the time-invariant factor. All community-level time-invariant factors including one used to construct the interaction terms are controlled for in the first difference specification.

#### Construction of variables

- Access to public river transportation—access to at least one public river boat (large river boat, small river boat, or speed boat) before the COVID-19 pandemic.
- Access to large river/small river/speed boat—access to at least one large river/small public river/speed boat before the COVID-19 pandemic.
- Active/inactive public river transportation—at least one/no large river boat, small public river boat, or speed boat during the previous 7 days at the time of interviews (baseline, follow-up).
- Active/inactive large river/small river/speed boats—at least one/no large river/small public river/speed boat during the previous 7 days at the time of interviews (baseline, follow-up).
- Active/frequent local private river transportation—at least one/five small personal boats during the previous day at the time of interviews (baseline, follow-up).
- Distance from city—River network distance^60^ from Iquitos or Pucallpa (closer one) to each community.
- Distance from district capital—River network distance^60^ from district capital (including one outside the study area) to each community. The analysis sample covered 21 districts.
- Distance from nearest market town—River network distance^60^ from the nearest market town out of 13 (including ones outside the study area) to each community.
- Distance from nearest community—River network distance^60^ from the nearest community out of all other communities covered in the community survey from each community.
- River order—A positive whole number to indicate the level of branching in a river system. We consider the rivers on which Iquitos and Pucallpa (the source of virus) are situated as order 1. Specifically, we consider the Amazon, Ucayali, and Marañón rivers as order 1 (Fig. 1), although the latter two are order 2 according to hydrology conventions. The Pastaza River joins the Marañón (not depicted) which becomes the Amazon together with the Ucayali. The Pastaza River is treated as order 2 in our definition.
- Forest—The proportion of land area in a 5 km buffer centered on the community in 2015 which was classified from Landsat imagery to be forest with CLASlite v3.2^61^.
- Floodplain soil—A proxy for young alluvial soils in floodplain. The proportion of land area in a 5 km buffer centered on the community that is underlain by Holocene parent material. Based on *La Carta Geológica Nacional Mapa Geológico del Peru* (1:100,000) published by INGEMMET (Instituto Geológico Minero y Metalúrgico, Lima); available on-line at: https://portal.ingemmet.gob.pe/web/guest/carta-geologica-nacional-escala-1-100-000. Simplified reclassification of INGEMMET’s ‘Soil_NAME’ variable, as soils being formed during the Holocene period (Qh), Pleistocene (Qp), or earlier (Tertiary).

Since most market towns are district capitals, we use either distance from district capital or distance from nearest market town as a predictor. We report results using distance from district capital; those using distance from nearest market town are similar.

## Supplementary Information

Supplementary Information.

## Data Availability

The data that support the findings of this study are available from the corresponding author upon reasonable request.

## References

[1] Walker PG (2020). The impact of COVID-19 and strategies for mitigation and suppression in low-and middle-income countries. Science.

[2] Brooks SK (2020). The psychological impact of quarantine and how to reduce it: rapid review of the evidence. The Lancet.

[3] World Bank. *Poverty and Shared Prosperity 2020: Reversals of Fortune*. (The World Bank, 2020).

[4] Egger, D. *et al.* Falling living standards during the COVID-19 crisis: Quantitative evidence from nine developing countries. *Sci. Adv.* **7**, eabe0997 (2021). 10.1126/sciadv.abe0997 PMC7864564 33547077

[5] Bundervoet T, Dávalos ME, Garcia N (2022). The short-term impacts of COVID-19 on households in developing countries: An overview based on a harmonized dataset of high-frequency surveys. World Dev..

[6] Josephson A, Kilic T, Michler JD (2021). Socioeconomic impacts of COVID-19 in low-income countries. Nat. Hum. Behav..

[7] Mobarak AM (2022). End COVID-19 in low-and middle-income countries. Science.

[8] Miguel E, Mobarak AM (2022). The economics of the COVID-19 pandemic in poor countries. Ann. Rev. Econ..

[9] Ranscombe P (2020). Rural areas at risk during COVID-19 pandemic. Lancet. Infect. Dis.

[10] Sitko N, Knowles M, Viberti F, Bordi D (2022). Assessing the Impacts of the COVID-19 Pandemic on the Livelihoods of Rural People: A Review of Evidence.

[11] Takasaki Y, Coomes OT, Abizaid C (2021). COVID-19 among Rural Peoples in the Peruvian Amazon: Policy Brief.

[12] Abizaid, C., Collado Panduro, L. Á. & Gonzales Egusquiza, S. Pobreza y Medios de Subsistencia en la Amazonía Peruana en Tiempos del COVID-19. *J. Lat. Am. Geogr.* **19**, 202–214 (2020).

[13] Taylor L (2021). Covid-19: Why Peru suffers from one of the highest excess death rates in the world. BMJ.

[14] Schwalb A, Seas C (2021). The COVID-19 pandemic in Peru: What went wrong?. Am. J. Trop. Med. Hyg..

[15] Calderon-Anyosa RJ, Bilal U, Kaufman JS (2021). Variation in non-external and external causes of death in Peru in relation to the COVID-19 lockdown. Yale J. Biol. Med..

[16] Chibnik, M. *Risky Rivers: The Economics and Politics of Flood Plain Farming in Amazonia*. (The University of Arizona Press, 1994).

[17] Balcan D (2009). Multiscale mobility networks and the spatial spreading of infectious diseases. Proc. Natl. Acad. Sci. USA.

[18] Ruan Z, Wang C, Hui PM, Liu Z (2015). Integrated travel network model for studying epidemics: Interplay between journeys and epidemic. Sci. Rep..

[19] Strano E, Viana MP, Sorichetta A, Tatem AJ (2018). Mapping road network communities for guiding disease surveillance and control strategies. Sci. Rep..

[20] Franch-Pardo I, Napoletano BM, Rosete-Verges F, Billa L (2020). Spatial analysis and GIS in the study of COVID-19. A review. Sci. Total Environ..

[21] Nicolelis MA, Raimundo RL, Peixoto PS, Andreazzi CS (2021). The impact of super-spreader cities, highways, and intensive care availability in the early stages of the COVID-19 epidemic in Brazil. Sci. Rep..

[22] Tatem AJ (2014). Integrating rapid risk mapping and mobile phone call record data for strategic malaria elimination planning. Malar. J..

[23] Tatem AJ, Smith DL (2010). International population movements and regional Plasmodium falciparum malaria elimination strategies. Proc. Natl. Acad. Sci. USA.

[24] Gross B (2020). Spatio-temporal propagation of COVID-19 pandemics. EPL (Europhys. Lett.).

[25] Tatem AJ (2014). Mapping population and pathogen movements. Int. Health.

[26] Wilder-Smith A, Freedman DO (2020). Isolation, quarantine, social distancing and community containment: pivotal role for old-style public health measures in the novel coronavirus (2019-nCoV) outbreak. J. Travel Med..

[27] Flaxman S (2020). Estimating the effects of non-pharmaceutical interventions on COVID-19 in Europe. Nature.

[28] Chinazzi M (2020). The effect of travel restrictions on the spread of the 2019 novel coronavirus (COVID-19) outbreak. Science.

[29] Howard J (2021). An evidence review of face masks against COVID-19. Proc. Natl. Acad. Sci. USA.

[30] Chibnik M (1991). Quasi-ethnic groups in Amazonia. Ethnology.

[31] Flores-Ramírez R (2021). A review of environmental risks and vulnerability factors of indigenous populations from Latin America and the Caribbean in the face of the COVID-19. Glob. Public Health.

[32] Mallard A, Pesantes MA, Zavaleta-Cortijo C, Ward J (2021). An urgent call to collect data related to COVID-19 and indigenous populations globally. BMJ Glob. Health.

[33] Montag D (2021). Healthcare of Indigenous Amazonian Peoples in response to COVID-19: marginality, discrimination and revaluation of ancestral knowledge in Ucayali, Peru. BMJ Glob. Health.

[34] Soto-Cabezas MG (2022). COVID-19 among Amazonian indigenous in Peru: mortality, incidence, and clinical characteristics. J. Public Health.

[35] Salonen M, Toivonen T, Cohalan J-M, Coomes OT (2012). Critical distances: Comparing measures of spatial accessibility in the riverine landscapes of Peruvian Amazonia. Appl. Geogr..

[36] Abizaid C, Coomes OT, Takasaki Y (2022). Lifeways and currents of change in the Peruvian Amazon: A 1000 km boat journey down the Ucayali River. Focus Geogr..

[37] Hallal PC (2020). SARS-CoV-2 antibody prevalence in Brazil: results from two successive nationwide serological household surveys. Lancet Glob. Health.

[38] Okyere I (2020). Physical distancing and risk of COVID-19 in small-scale fisheries: a remote sensing assessment in coastal Ghana. Sci. Rep..

[39] Abdoul-Azize HT, El Gamil R (2021). Social protection as a key tool in crisis management: learnt lessons from the COVID-19 pandemic. Global Social Welfare.

[40] Lowe, C., McCord, A. & Beazley, R. *National Cash Transfer Responses to Covid-19*. Working paper No. 610 (ODI, London, 2021).

[41] Gentilini U (2022). Social protection and jobs responses to COVID-19: A real-time review of country measures.

[42] Pajuelo-Reyes C (2021). Epidemiological analysis of COVID-19 cases in native Amazonian Communities from Peru. Epidemiologia.

[43] Verhagen LM (2020). COVID-19 response in low-and middle-income countries: Don’t overlook the role of mobile phone communication. Int. J. Infect. Dis..

[44] Takasaki Y, Coomes OT, Abizaid C (2022). COVID-19 information and self-protective behaviors among rural communities in tropical forests. BMC Public Health.

[45] Yaqub O, Castle-Clarke S, Sevdalis N, Chataway J (2014). Attitudes to vaccination: A critical review. Soc. Sci. Med..

[46] Salmon DA, Dudley MZ, Glanz JM, Omer SB (2015). Vaccine hesitancy: causes, consequences, and a call to action. Vaccine.

[47] Solís Arce JS (2021). COVID-19 vaccine acceptance and hesitancy in low-and middle-income countries. Nat. Med..

[48] Rodriguez-Morales AJ, Franco OH (2021). Public trust, misinformation and COVID-19 vaccination willingness in Latin America and the Caribbean: today’s key challenges. Lancet Region. Health – Am..

[49] Gutiérrez-Zevallos JD, Espíritu-Martínez LB (2021). COVID-19: vaccination in a developing country. J. Public Health.

[50] Berkouwer, S. B. *et al.**Money or Power? Financial Infrastructure and Optimal Policy*. NBER Working Paper No. 29086 (National Bureau of Economic Research, Cambridge, 2021).

[51] Londoño-Vélez J, Querubin P (2022). The impact of emergency cash assistance in a pandemic: Experimental evidence from Colombia. Rev. Econ. Stat..

[52] Sardinha DM (2021). Risk factors associated with the severity of COVID-19 in a region of the Brazilian Amazon. Sci. Rep..

[53] INEI. *Perú: Estimaciones y Proyecciones de Población Urbana y Rural por Sexo y Edades Quinquenales, según Departamento, 2000–2015*. Boletín Especial 19 (Instituto Nacional de Estadística e Informática, Lima, 2015).

[54] Kvist LP, Gram S, Cácares AC, Ore IB (2001). Socio-economy of flood plain households in the Peruvian Amazon. For. Ecol. Manag..

[55] Takasaki Y, Barham BL, Coomes OT (2001). Amazonian peasants, rain forest use, and income generation: The role of wealth and geographical factors. Soc. Nat. Resour..

[56] Coomes OT, Takasaki Y, Abizaid C, Arroyo-Mora JP (2016). Environmental and market determiants of economic orientation among rain forest communities: Evidence from a large-scale survey in western Amazonia. Ecol. Econ..

[57] Smith RC, Benavides M, Pariona M, Tuesta E (2003). Mapping the past and the future: Geomatics and indigenous territories in the Peruvian Amazon. Hum. Organ..

[58] Benavides, M. *Atlas de comunidades nativas y áreas naturales protegidas del nordeste de la Amazonía peruana*. (Instituto del Bien Común, 2010).

[59] Banerjee, A. *et al.**Messages on COVID-19 prevention in India increased symptoms reporting and adherence to preventive behaviors among 25 million recipients with similar effects on non-recipient members of their communities*. NBER Working Paper No. 27496 (National Bureau of Economic Research, Cambridge, 2020).

[60] Webster K, Arroyo-Mora JP, Coomes OT, Takasaki Y, Abizaid C (2016). A cost path and network analysis methodology to calculate distances along a complex river network in the Peruvian Amazon. Appl. Geogr..

[61] Asner GP, Knapp DE, Balaji A, Páez-Acosta G (2009). Automated mapping of tropical deforestation and forest degradation: CLASlite. J. Appl. Remote Sens..

