# Supplementary material for: COVID-19 contagion across remote communities in tropical forests
Source: Sci Rep. 2022 Dec 1;12:20727. doi: 10.1038/s41598-022-25238-7 (PMC9713114; doi:10.1038/s41598-022-25238-7)
Supplement: Supplementary file 1 — Supplementary Information. [file 41598_2022_25238_MOESM1_ESM.pdf]

Supplementary Information

COVID-19 contagion across remote communities in tropical forests

Yoshito Takasaki*, Christian Abizaid, Oliver T. Coomes

*Corresponding author. Email: takasaki@e.u-tokyo.ac.jp

This Supplementary Information includes:

- Supplementary Notes 1 to 9
- Supplementary References (1-4)
- Supplementary Figures 1 to 15
- Supplementary Tables 1 to 7

Supplementary Notes

Supplementary Note 1: Mortality

By the time of the baseline survey, 12% of communities experienced any death caused by COVID-19, including suspected ones (88 deaths in total). According to the regression analysis based on equation (1), mortality was more common in communities far from cities and mestizo communities; it was not related to the availability of health facility (Supplementary Table 1).

Supplementary Note 2: Robustness check

To assess the robustness of our OLS estimates based on equation (1) to omitted variable bias, we employ the coefficient stability test against omitted variable bias in the regression framework[1]. For each predictor whose point estimate is statistically significant at least at a 10% significant level reported in Fig. 3, we consider the selected predictor as a treatment variable and other predictors as covariates. Following [1], we set the R-squared value from the regression with a complete set of covariates including unobservable ones (*Rmax*) at 1.3 times the R-squared value from the regression with observable covariates. We calculate the degree of selection on unobservables relative to observable covariates (denoted by δ) for which the OLS estimate for the selected predictor (denoted by β) becomes zero. Following [1], we consider $\delta = 1$ as a cutoff: a value of $\delta = 1$ suggests that the unobservables would need to be as important as the observables to produce a treatment effect of zero.

The results are reported in Supplementary Tables 1 and 3. For most estimates in Fig. 3, δ is greater than 1 or almost 1; as exceptions, δ is smaller than 0.8 for (1) distance from city for the mitigation of COVID-19 by the time of the baseline survey, and (2) inactive public river transportation for case incidence at the baseline. These results suggest that most regression estimates are qualitatively robust to omitted variable bias.

Supplementary Note 3: Spatial distributions

Public river transportation was less available in the Pastaza (Supplementary Fig. 10A). Inactivated public river transportation at the follow-up was common in the Amazon and activated transportation at the follow-up was common in upper and lower Napo.

Communication access was more limited in the Department of Loreto than in Ucayali (Supplementary Fig. 10B). This means that we could cover relatively more communities with no communication access in Loreto than Ucayali.

Preventive measures (index) were strong in the Napo before the baseline survey and at the follow-up survey and became weaker in the Amazon and the lower Ucayali over time (Supplementary Figs. 11A, 11B). Social restrictions (index) were strong in the Napo at the baseline and follow-up surveys and became stronger in the Napo, the lower reach of the Pastaza, and the middle reach of the Lower Ucayali over time (Supplementary Fig. 11C, Fig. 3D). These spatial patterns are consistent with the distinct evolution by indigeneity: self-protective behaviors became relatively stronger in Indigenous communities than in mestizo communities (Supplementary Figs. 7B, 7D).

Supplementary Note 4: Predictors of public river transportation

We regress access to public river transportation on the same predictors as the main analysis except for active and inactive public river transportation using equation (1) (Supplementary Fig. 6B). Public transportation (large and small river boats) was more available

in larger communities with higher demand for cargo and passengers; the reverse holds true for speed boats. Large river boats were more available in communities far from cities, reflecting their importance in remote communities (see Supplementary Table 6 for other predictors).

Next, we regress active public river transportation on the same predictors plus access to public river transportation (Supplementary Fig. 6B). Given access to public river transportation, active transportation (large river boats) was less common in larger communities at the baseline, possibly due to concerns about the spread of virus. Active transportation (any mode) was more common in communities far from cities at both baseline and follow-up (with the exception of large river boats at the follow-up) (see Supplementary Table 6 for other predictors).

Supplementary Note 5: Timing of initial spread as predictors

We repeat the main analysis for COVID-19 mitigation by the time of the baseline survey, for case incidence at the time of the baseline survey, for case incidence at the follow-up survey, and for mitigation, persistence, and spread between the baseline and follow-up surveys (Figs. 2A, B) using equation (1) by controlling for the timing of initial spread (by March, April, May, and June) as additional predictors. Since initial spread is an endogenous variable, the analysis captures correlations. The timing of initial spread was not differentially related to any outcomes (Supplementary Figs. 12A-12F). Thus, communities where early spread occurred did not necessarily mitigate the contagion earlier. Mortality by the time of the baseline survey was common in communities where initial spread occurred during the first wave in April and May (Supplementary Fig. 12G).

Supplementary Note 6: Individual self-preventive behavioral measures

The evolution of individual preventive measures varied between Indigenous and mestizo communities (Supplementary Fig. 13). Handwashing and use of a mask were less common in Indigenous communities than in mestizo communities before the baseline. They became more common only in Indigenous communities at the follow-up but were still less common than mestizo communities. Social distancing measures became less common over time and somewhat more so in mestizo communities. As a result, although preventive measure index was greater in mestizo communities than Indigenous communities before the baseline, the converse holds true at the follow-up (Supplementary Figs. 7B, 7C).

Individual social restriction measures became more (less) common in Indigenous (mestizo) communities over time (albeit small difference) (Supplementary Fig. 13). The evolution of social restriction index was consistent (Supplementary Figs. 7B, 7C).

Supplementary Note 7: Effectiveness of individual self-preventive behavioral measures

We estimate impacts of individual self-preventive behavioral measures on the mitigation and spread of COVID-19 using equation (2). For each individual behavioral measure (binary indicator), we consider activated and inactivated measure in the same way as public river transportation. Although most results are statistically weak and the signs of point estimates vary, their patterns are largely consistent with the index results (Supplementary Fig. 14). Activated handwashing, avoiding gatherings, and no gatherings for church service increased mitigation and/or reduced spread. Most inactivated social distancing measures increased spread and inactivated secondary school closure reduced mitigation.

Supplementary Note 8: Public river transportation modes

We repeat the main analysis using equation (1) for three modes of public river transportation and three modes of communication separately (Supplementary Fig. 15). On one hand, COVID-19 spread was delayed in communities with inactive large river boats through time by almost the same magnitude found for inactive public river transportation. Active local river transportation (small personal boats) was more common in communities with large and small public river boats at both baseline and follow-up. Frequent local river transportation was more common in communities with large river boats at both baseline and follow-up. These results for large and small boats for both passengers and cargo buttress our finding that COVID-19 spread through river transportation was driven by market access. On the other hand, the probability of receiving government cash assistance before the baseline survey was lower in communities with inactive large river boats by almost the same magnitude (43%) found for inactive public river transportation. This suggests that the spread through social assistance occurred mainly through large river boats. Large river boats have been found to contribute to the spread of the dengue vector, *Aedes aegypti*, near Iquitos because they provided suitable mosquito habitat[2].

We repeat the main analysis using equation (2) for three modes of public river transportation separately (Supplementary Fig. 8D). Inactivated large river boats reduced the probability of spread at almost the same magnitude (by 0.15) found for inactivated public river transportation. Activated large river boats reduced the probability of mitigation at almost the same magnitude (by 0.21) found for activated public river transportation. These results suggest that COVID-19 evolution was shaped mainly by large river boats.

Supplementary Note 9: Social assistance and return migration

In contrast to government cash assistance, government food assistance which was delivered to communities (78% of them had received it before the baseline survey) was less common in communities with active public river transportation (by 0.2), reflecting the state's targeting of such assistance to communities with limited market access (Supplementary Table 3). Food assistance at the time of the follow-up survey was uncommon. The provision of masks, sanitizer, soap, and medicines was almost nonexistent and non-governmental institutions' support was very limited[3].

Return migration from cities and towns to rural communities has been identified as a common coping response during the pandemic[4]. Return migration was observed in 45% of communities at the time of the follow-up survey. Return migration to reduce infection risk and to obtain help from people in kin-based communities (families, relatives, friends) was more common (by 12% and 9%, respectively) in communities with active public river transportation; this pattern was not observed for return migration to secure food, livelihoods, or to maintain social life (Supplementary Table 7). These results suggest that return migration to cope with the pandemic among people in cities and towns using public river transportation also contributed to the spread of COVID-19.

Supplementary References

1 Oster, E. Unobservable selection and coefficient stability: Theory and evidence. *Journal of Business & Economic Statistics* **37**, 187-204 (2019).

2 Guagliardo, S. A. *et al.* River boats contribute to the regional spread of the dengue vector Aedes aegypti in the Peruvian Amazon. *PLoS Neglected Tropical Diseases* **9**, e0003648 (2015).

3 Takasaki, Y., Coomes, O. T. & Abizaid, C. COVID-19 among rural peoples in the Peruvian Amazon: Policy brief. (University of Tokyo, Tokyo, 2021).
4 DeFries, R. *et al.* Post-lockdown spread of COVID-19 from cities to vulnerable forest-fringe villages in Central India. *Current Science* **119**, 52-58 (2020).

Supplementary Figures

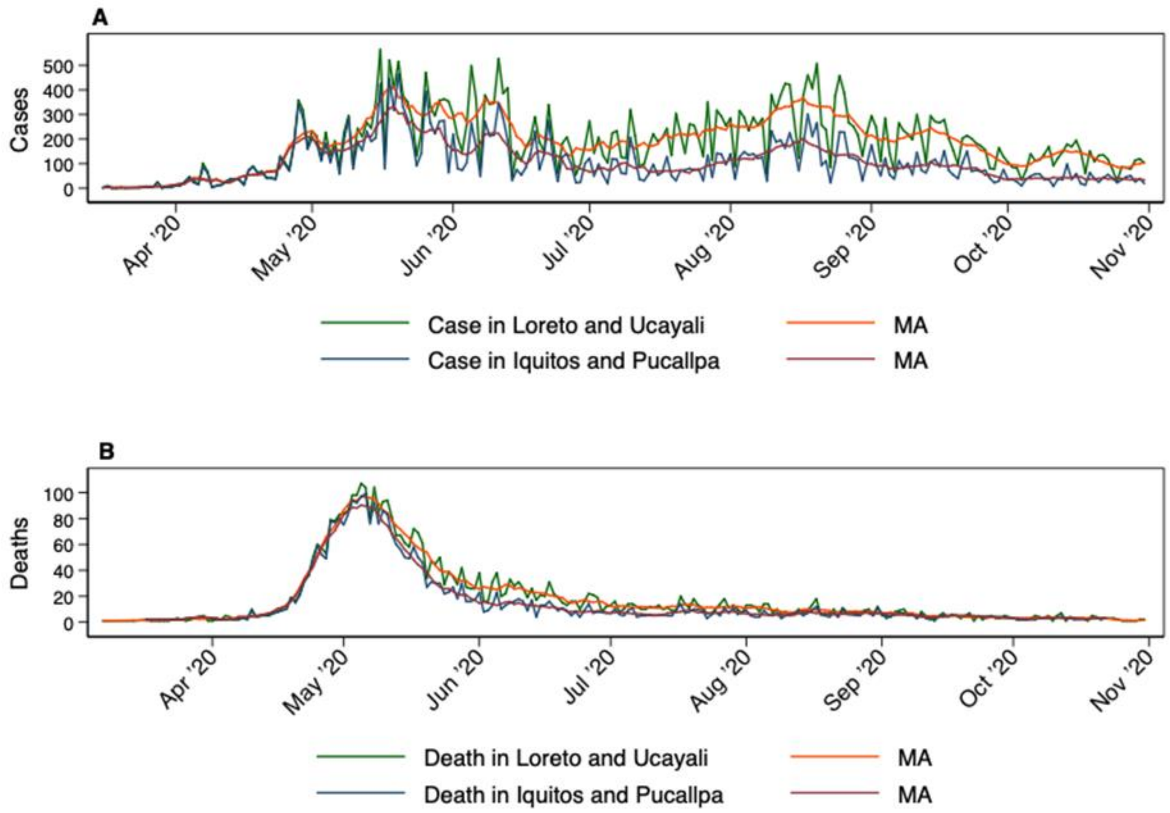

Supplementary Fig. 1. Regional trends of COVID-19 prevalence and mortality. Daily confirmed cases of COVID-19 (**A**) and daily mortality due to COVID-19 (**B**) in Loreto and Ucayali, and Iquitos (Iquitos, Punchana, Belén, San Juan Bautista Districts) and Pucallpa (Callería, Yarinacoha, Manantay Districts) with symmetric 7-day moving average (MA). Source: Peruvian Ministry of Health (Ministerio de Salud, MINSA), https://www.datosabiertos.gob.pe/group/datos-abiertos-de-covid-19, accessed June 16, 2021.

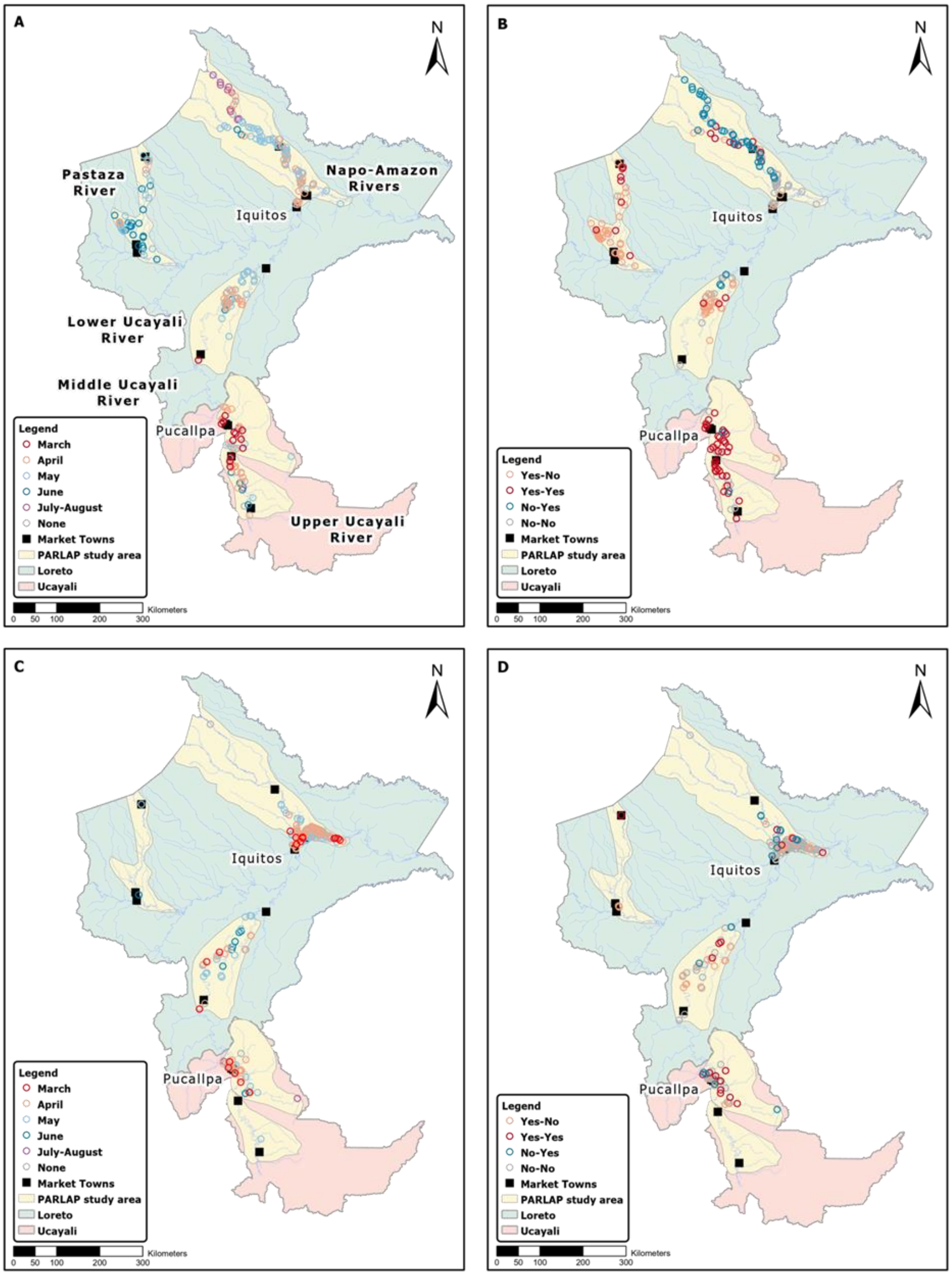

Supplementary Fig. 2. COVID-19 spread and evolution across communities by indigeneity. First COVID-19 case in 2020 in Indigenous communities (**A**), COVID-19 evolution between the baseline and follow-up surveys in Indigenous communities (for example, Yes-No means any case at the baseline and no case at the follow-up) (**B**), first COVID-19 case in 2020 in mestizo communities (**C**), COVID-19 evolution between the baseline and follow-up surveys in mestizo communities (**D**). In A and C, June includes two communities which experienced first case in July and July-August means first case which occurred between the baseline and follow-up surveys.

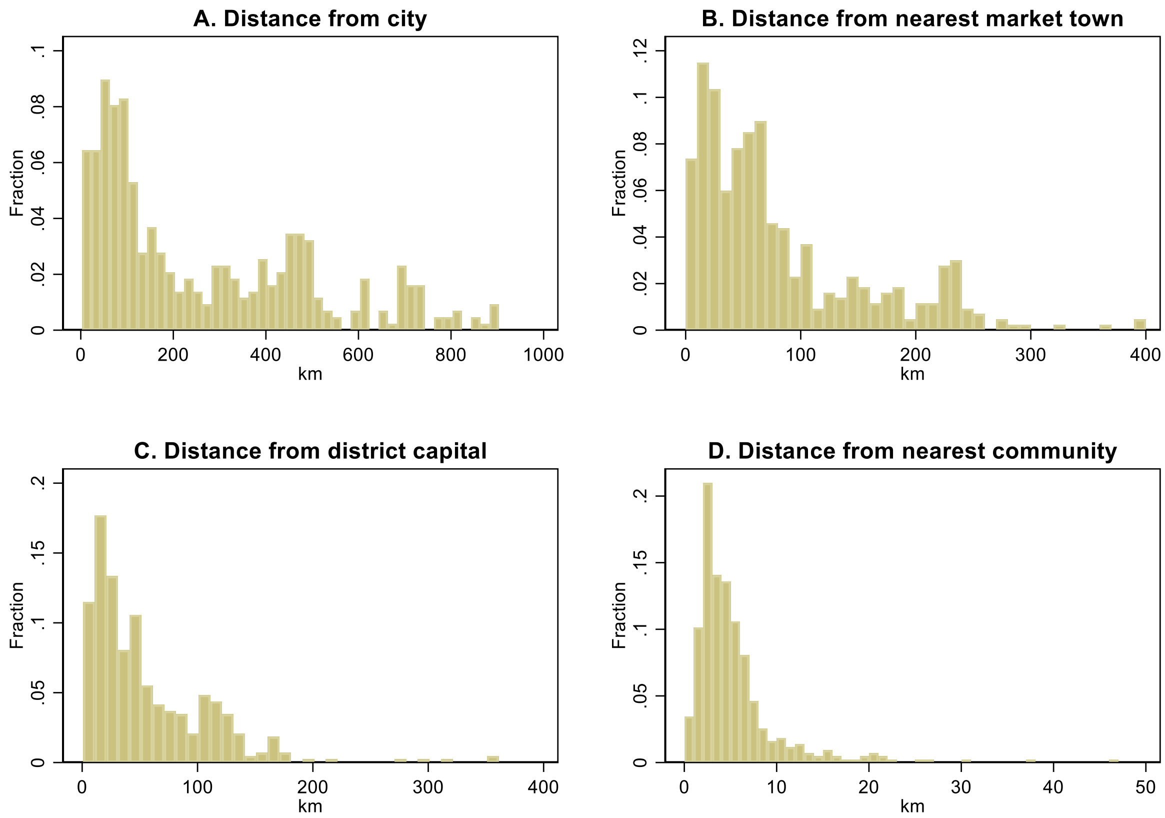

Supplementary Fig. 3. Histogram plots of distance. Histogram plots of distance from city, year of community establishment, and year of household formation. Fractions are shown. River network distance from city (Iquitos or Pucallpa) (**A**), nearest market town (**B**), district capital (**C**), and nearest community in the community survey sample (**D**). One community with distance from nearest community greater than 100 km is dropped in D for exposition.

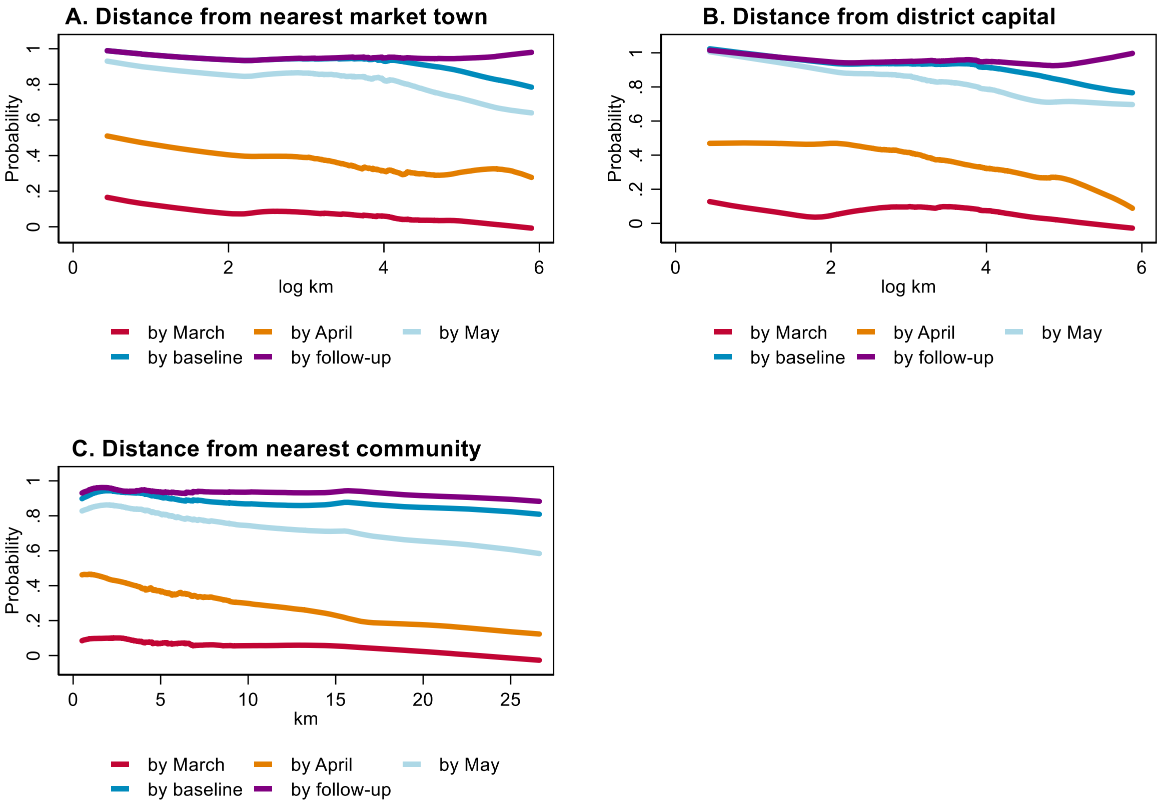

Supplementary Fig. 4. Predicted first COVID-19 case. Nonparametric relationship of first case with distance from nearest market town (**A**), district capital (**B**), and nearest community in the community survey sample (**C**). Lowess (locally weighted scatterplot smoothing) smoothers are shown. Communities whose nearest market town is Iquitos or Pucallpa are dropped in A and communities in Callería District whose capital is Pucallpa are dropped in B. The following communities are also dropped for exposition: two communities with log distance from nearest market town smaller than 0.4 km in A; one community with log distance from district capital smaller than 0.4 km in B; and five communities with distance from nearest community smaller than 0.5 km or greater than 30 km in C.

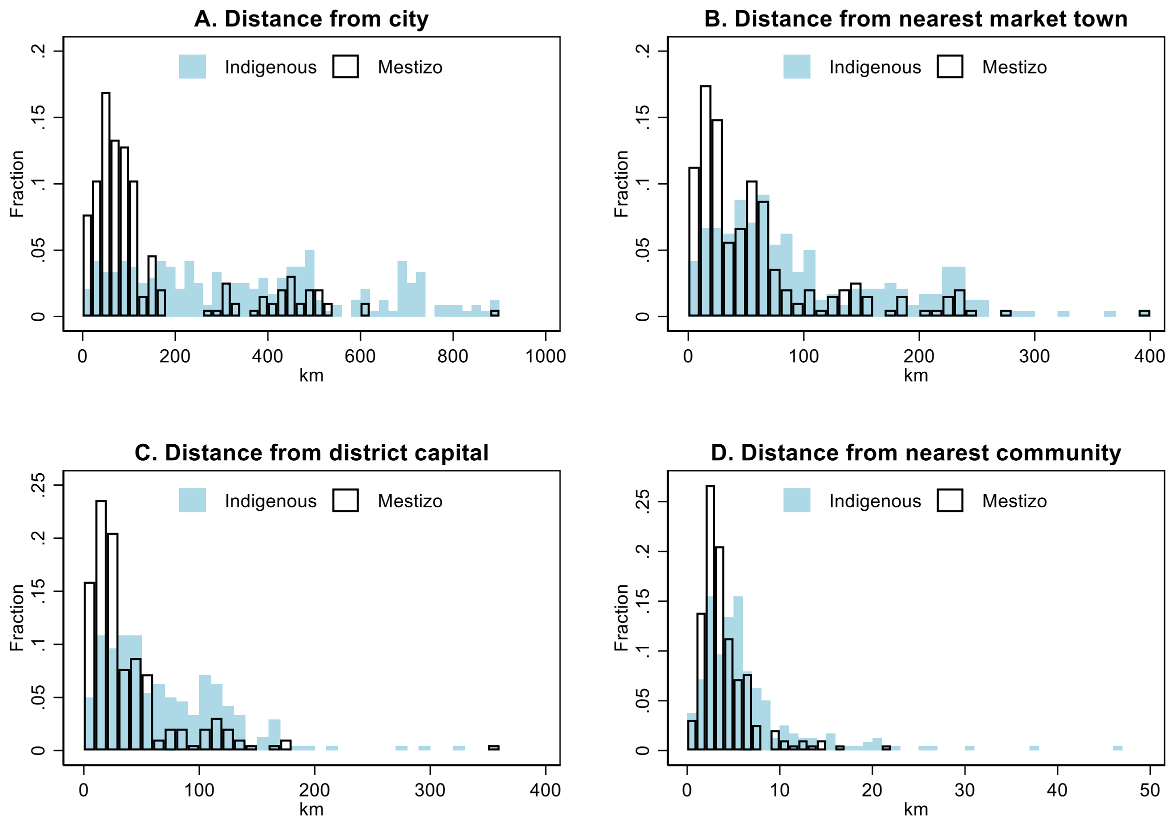

Supplementary Fig. 5. Histogram plots of distance by indigeneity. Fractions are shown. River network distance from city (Iquitos or Pucallpa) (**A**), nearest market town (**B**), district capital (**C**), and nearest community in the community survey sample (**D**). One community with distance from nearest community greater than 100 km is dropped in D for exposition.

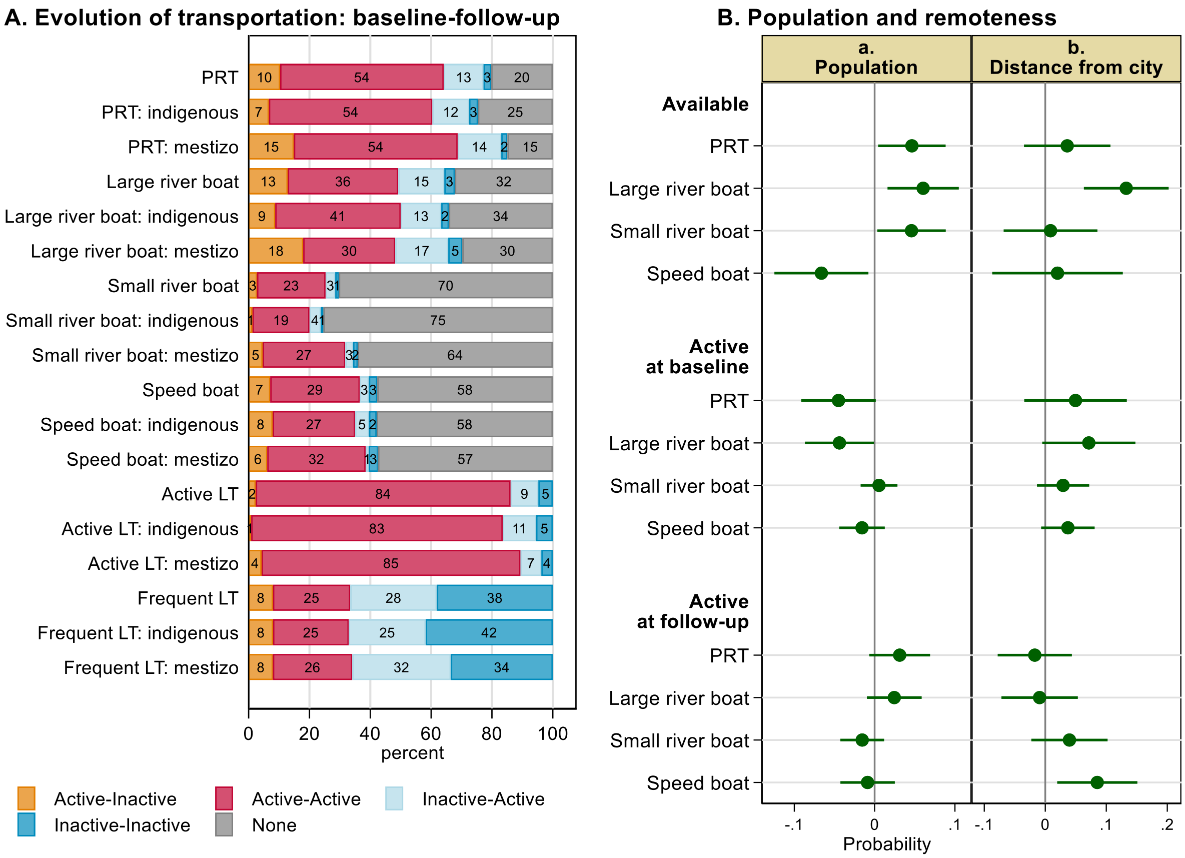

Supplementary Fig. 6. River transportation. Access to public river transportation (PRT) and evolution of PRT and local private river transportation (LT) between the baseline and follow-up surveys (for example, Active-Inactive means active PRT at the baseline and inactive PRT at the follow-up) (**A**), and the estimated predictors – population (log) and distance from city (log km) – of access to PRT (0/1) and active PRT at the baseline and follow-up (0/1) with 95% confidence intervals based on robust standard errors (**B**). See 'Empirical design' in Methods for the construction of transportation variables. Full regression results in B are reported in Supplementary Table 6.

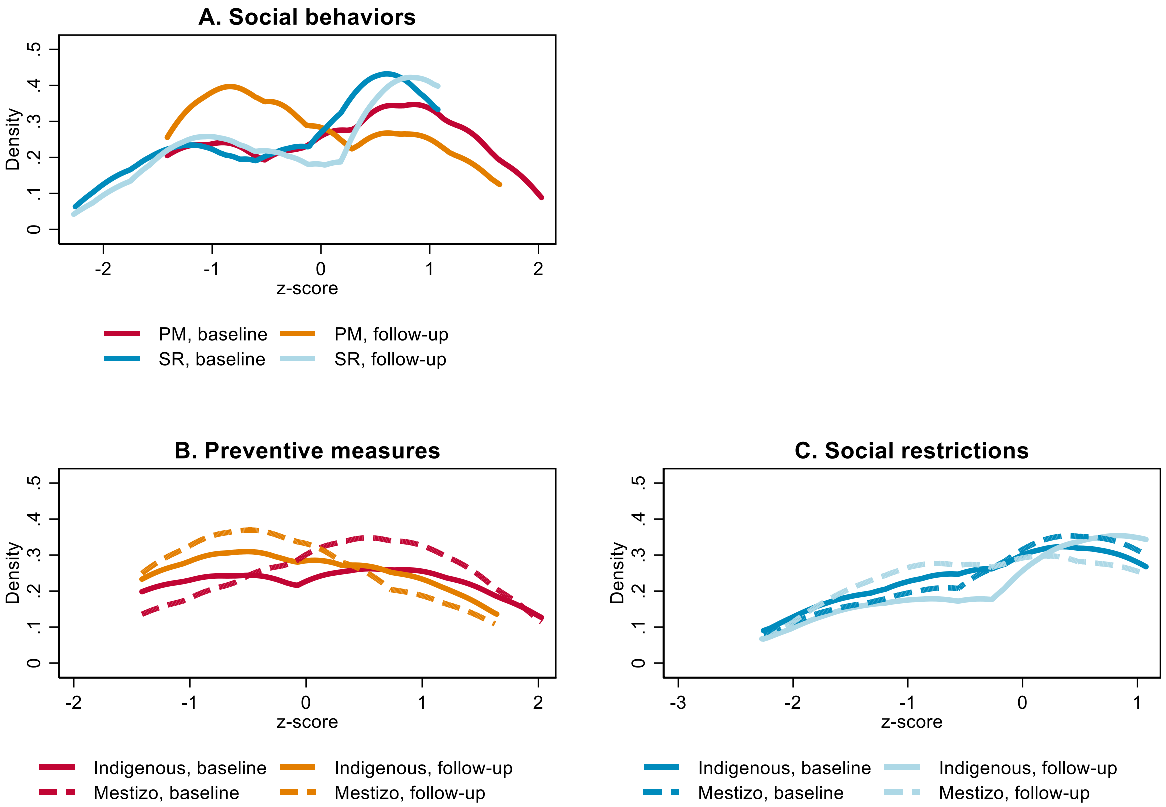

Supplementary Fig. 7. Distribution of social behaviors. The estimated densities of preventive measure (PM) and social restriction (SR) indices (z-score) in the whole sample (**A**), preventive measure index by indigeneity (**B**), and social restriction index by indigeneity (**C**).

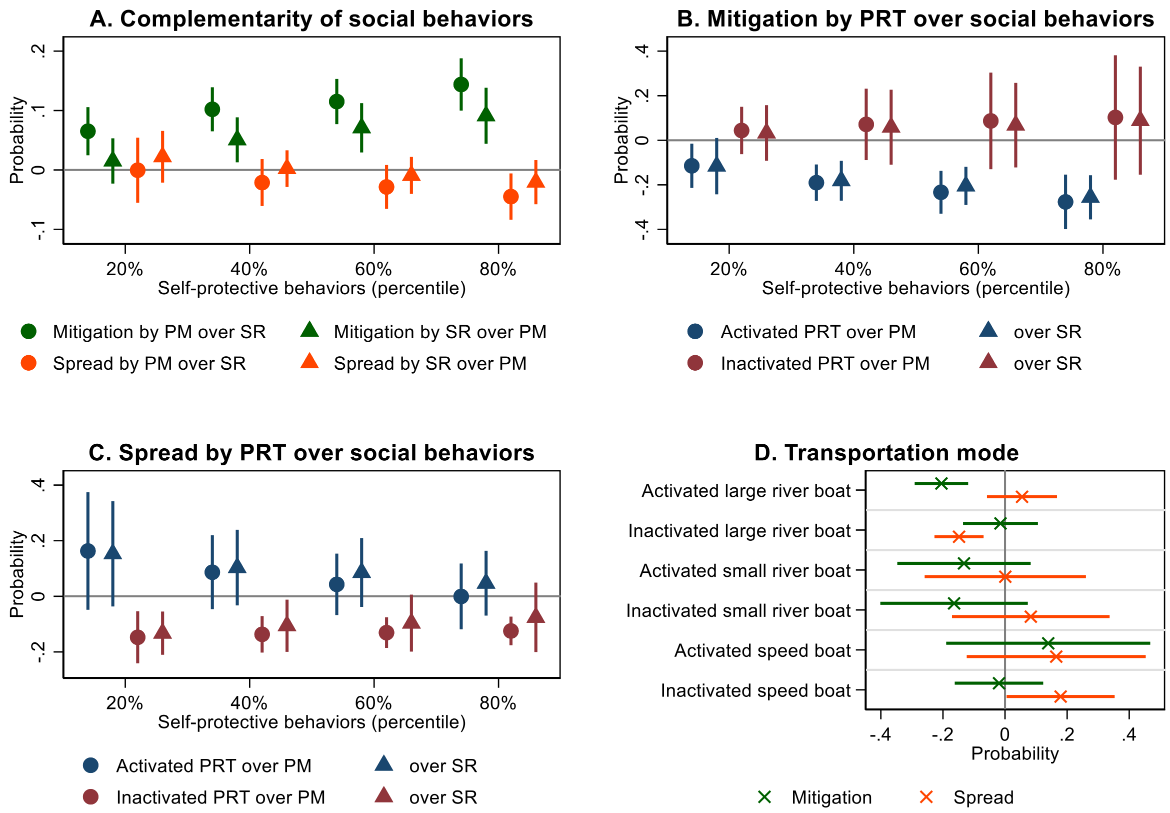

Supplementary Fig. 8. Complementarity and transportation mode. The estimated impacts of a change in preventive measures (PM) (z-score) on COVID-19 mitigation and spread between the baseline and follow-up surveys (0/1) over social restrictions (SR) (percentile) and the estimated impacts of a change in SR (z-score) on mitigation and spread (0/1) over PM (percentile) (**A**), the estimated impacts of activated and inactivated public river transportation (PRT) on mitigation over PM and SR (percentile) (**B**), the estimated impacts of activated and inactivated PRT on spread over PM and SR (percentile) (**C**), and the estimated impacts of activated and inactivated PRT modes on mitigation and spread (**D**), with 95% confidence intervals based on robust standard errors.

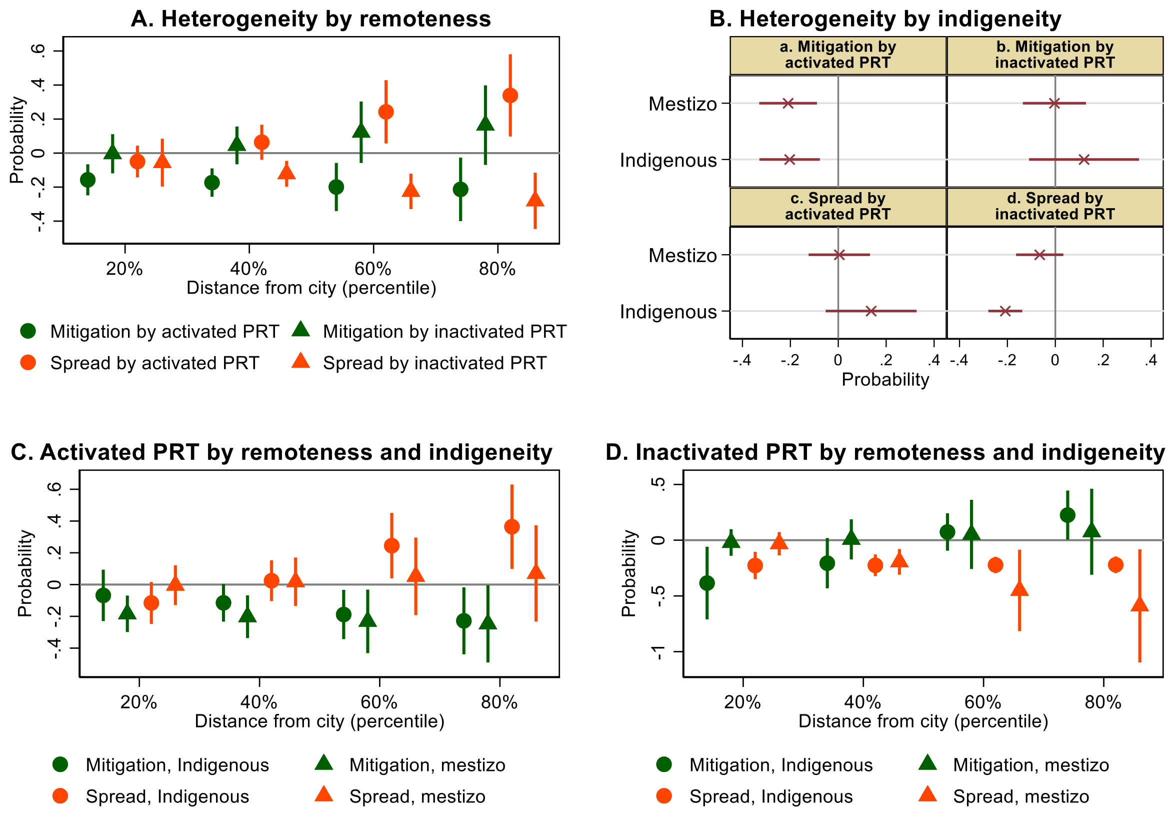

Supplementary Fig. 9. Transportation restrictions. The estimated impacts of activated and inactivated public river transportation (PRT) on COVID-19 mitigation and spread between the baseline and follow-up surveys (0/1) over distance from city (percentiles) (**A**) and by indigeneity (**B**), and the estimated impacts of activated (**C**) and inactivated (**D**) PRT on mitigation and spread (0/1) over distance from city (percentiles) and by indigeneity, with 95% confidence intervals based on robust standard errors.

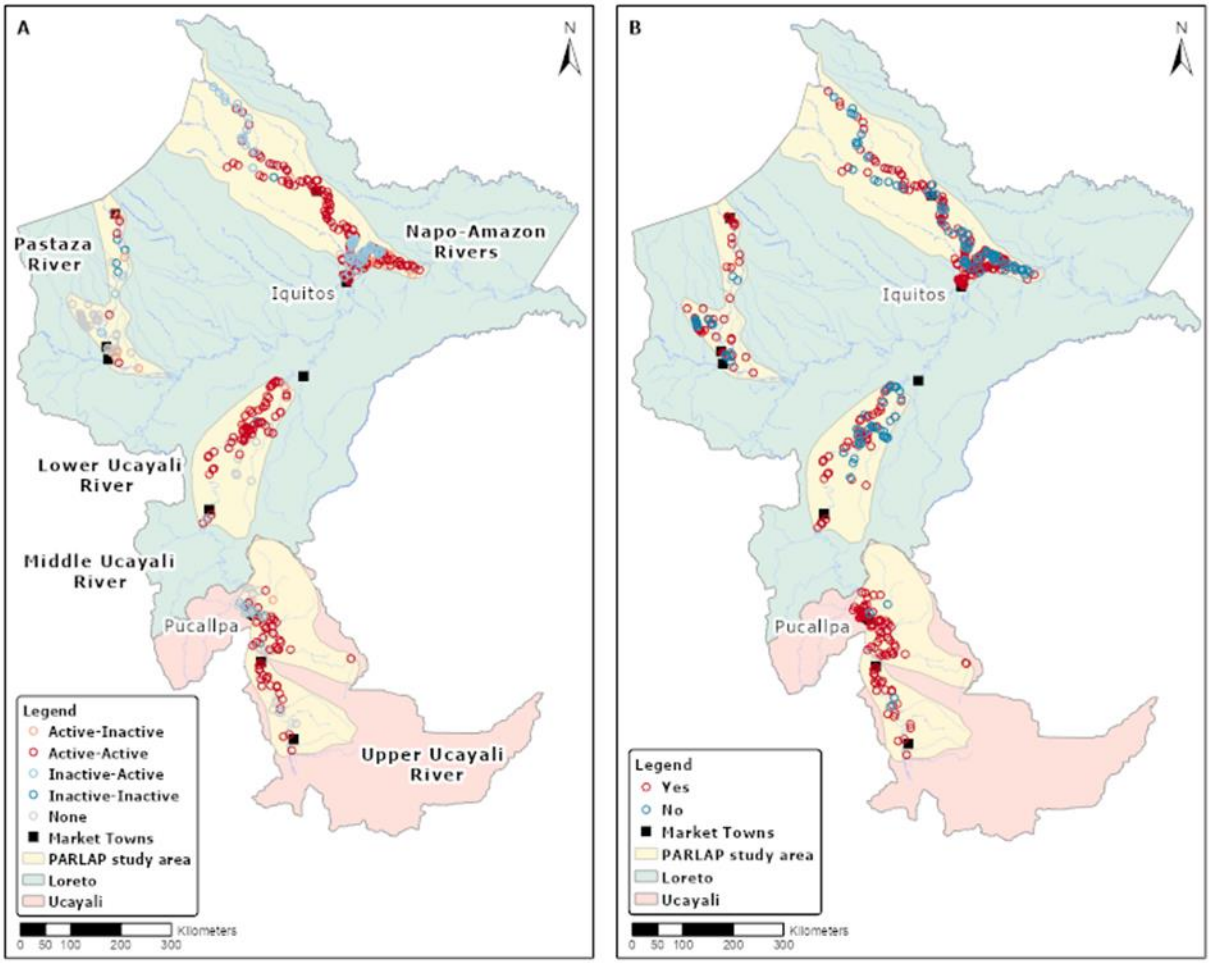

Supplementary Fig. 10. Public river transportation and communication access across communities. Access to public river transportation (PRT) and evolution of PRT between the baseline and follow-up surveys (for example, Active-Inactive means active PRT at the baseline and inactive PRT at the follow-up) (**A**) and communication access (**B**).

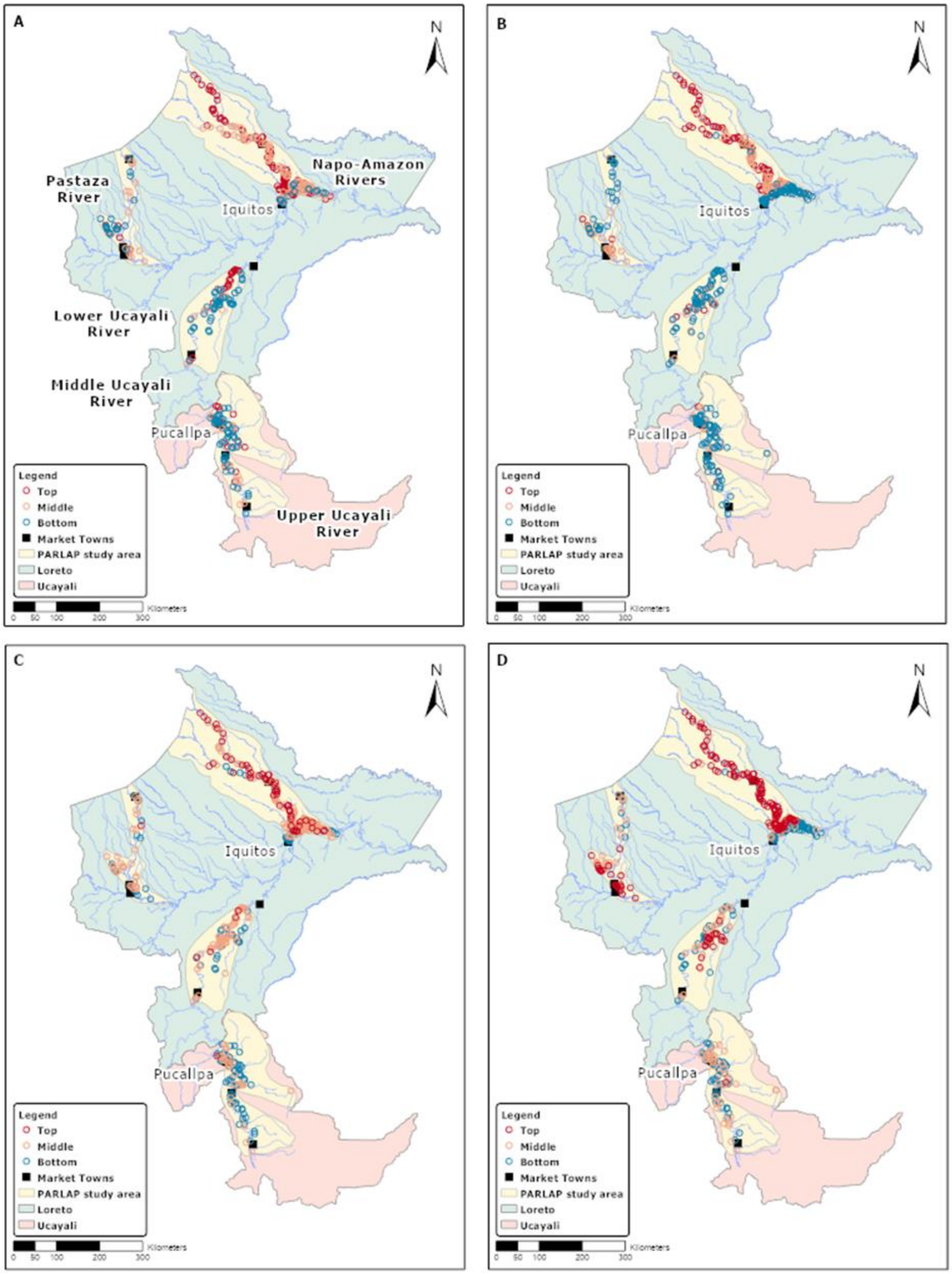

Supplementary Fig. 11. Social behaviors across communities. Preventive measures (z-score) before the baseline survey (tercile) (**A**) and at the follow-up survey (tercile) (**B**), social restrictions (z-score) at the baseline survey (tercile) (**C**) and at the follow-up survey (33.3 percentile and natural break) (**D**).

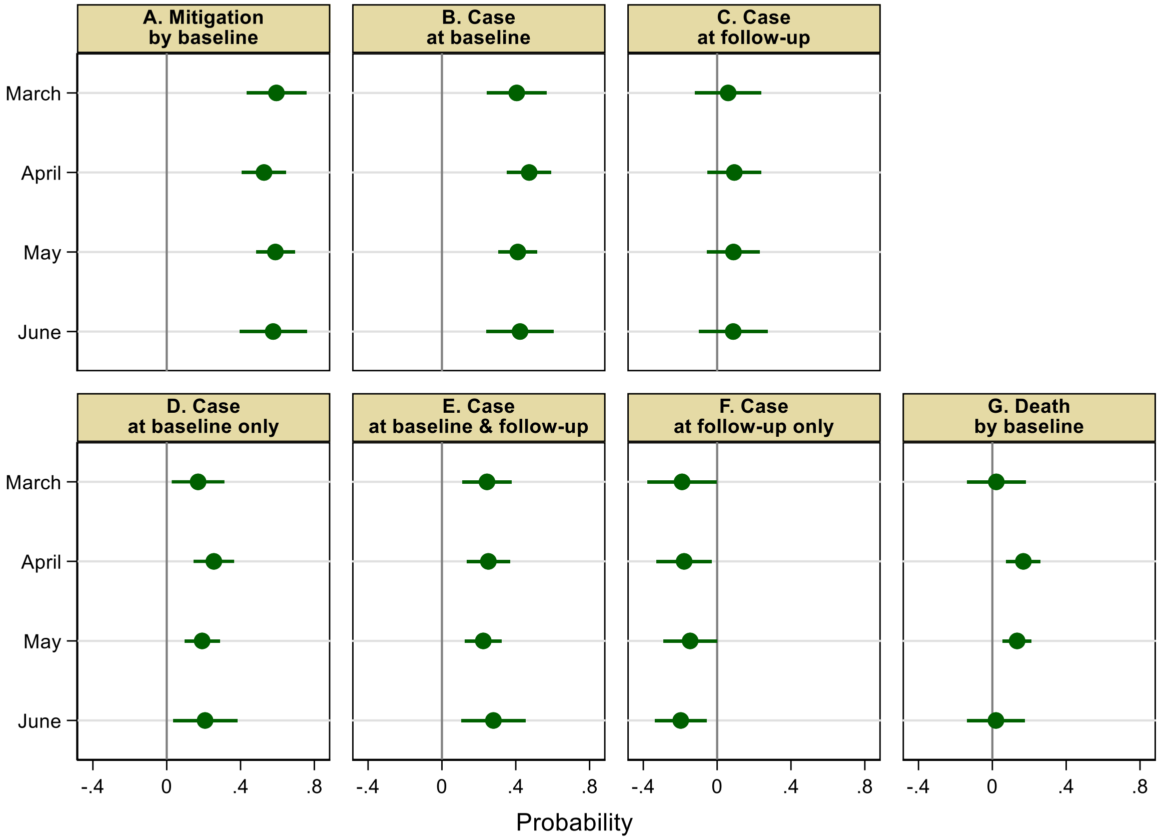

Supplementary Fig. 12. Timing of initial COVID-19 spread as predictors. The estimated correlations of the timing of first COVID-19 case in March, April, May, and June (0/1) with mitigation by the baseline survey (0/1) (**A**), case incidence at the baseline survey (0/1) (**B**), case incidence at the follow-up survey (0/1) (**C**), case incidence at the baseline survey but not follow-up survey (mitigation between the surveys) (0/1) (**D**), case incidence at both baseline and follow-up surveys (persistence between the surveys) (0/1) (**E**), case incidence at the follow-up survey but not baseline survey (spread between the surveys) (0/1) (**F**), and COVID-19 death by the baseline survey (0/1) (**G**), with 95% confidence intervals based on robust standard errors. June includes two communities which experienced first case in July.

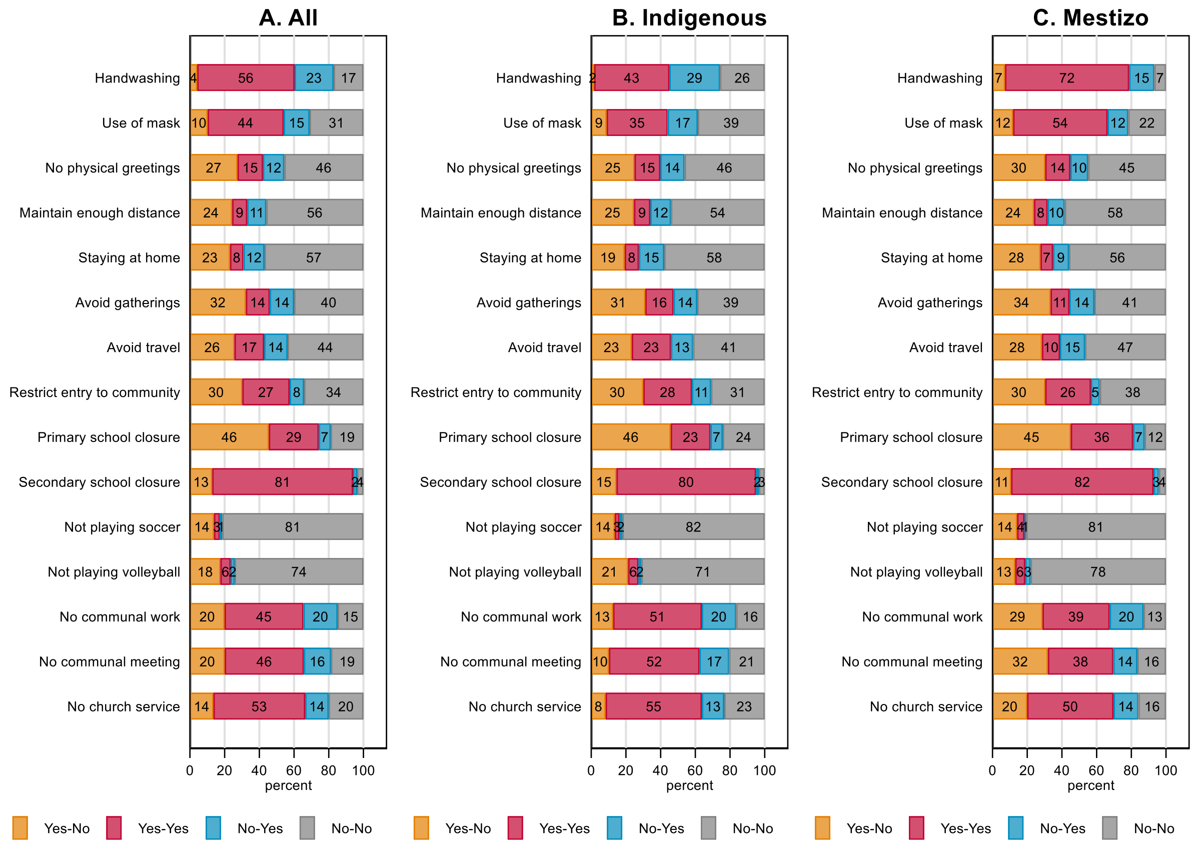

Supplementary Fig. 13. Individual self-preventive behavioral measures. Evolution of individual self-preventive behavioral measures between the baseline and follow-up surveys (for example, Yes-No means adoption at the baseline and no adoption at the follow-up) in the whole sample (**A**), among Indigenous communities (**B**), and among mestizo communities (**C**).

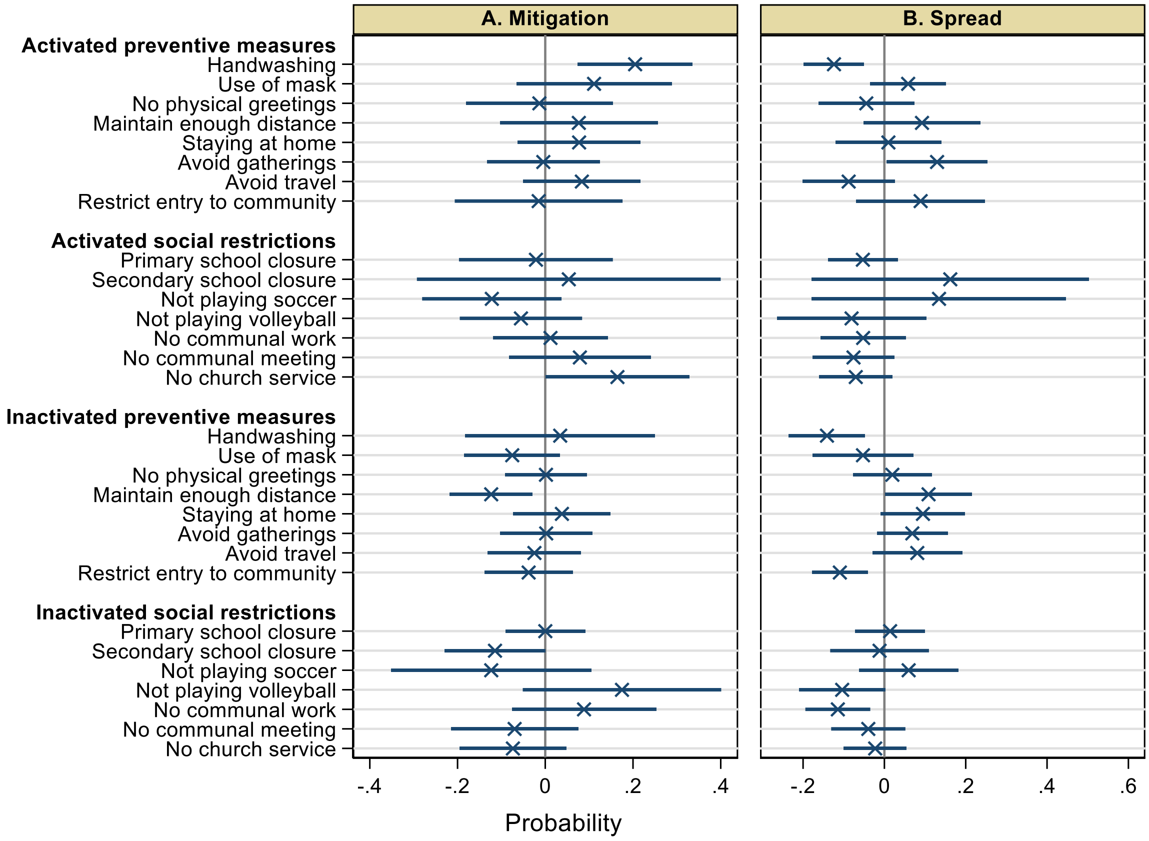

Supplementary Fig. 14. Effectiveness of individual self-protective behavioral measures. The estimated impacts of activated and inactivated individual self-protective behavioral measures on COVID-19 mitigation (**A**) and spread (**B**) between the baseline and follow-up surveys (0/1) with 95% confidence intervals based on robust standard errors.

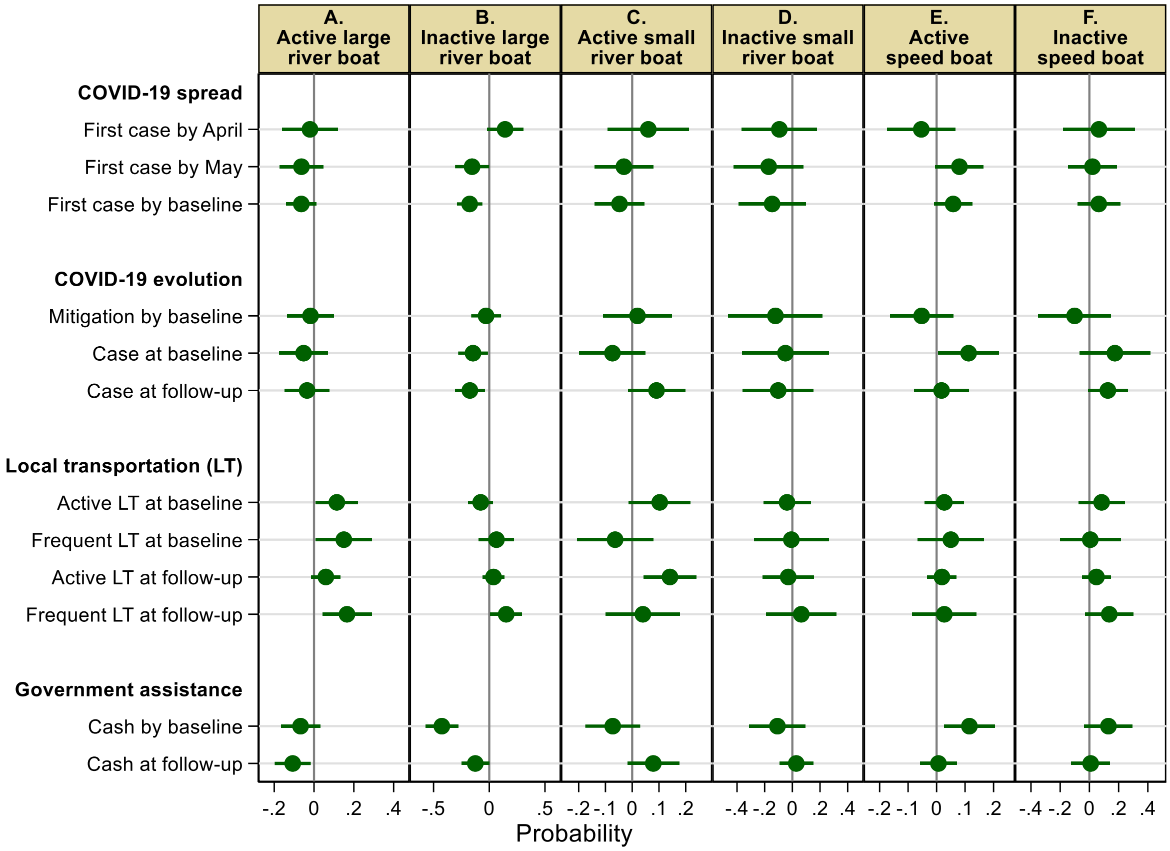

Supplementary Fig. 15. Transportation mode. The estimated impacts of active large river boat (0/1) (**A**), inactive large river boat (0/1) (**B**), active small public river boat (0/1) (**C**), inactive small public river boat (0/1) (**D**), active speed boat (0/1) (**E**), and inactive speed boat (0/1) (**F**) on COVID-19 spread and evolution, local private river transportation (LT), and government cash assistance (0/1), with 95% confidence intervals based on robust standard errors.

Supplementary Tables

Supplementary Table 1. OLS regression estimates for COVID-19 spread, evolution, and mortality

	First COVID-19 case by April (0/1) (1)	May (0/1) (2)	baseline (0/1) (3)	follow-up (0/1) (4)	Mitigation by baseline (0/1) (5)	Case at baseline (0/1) (6)	Case at follow-up (0/1) (7)	COVID-19 death by baseline (0/1) (8)
Distance from city (log km)	-0.161***	-0.108**	-0.0873*	0.0236	-0.114**	0.0252	0.203***	0.0724**
	(0.0505)	(0.0506)	(0.0488)	(0.0401)	(0.0519)	(0.0453)	(0.0453)	(0.0304)
Active public river transportation (PRT) (0/1)	-0.00259	-0.00318	-0.0376	-0.0367	-0.0227	-0.0198	0.0286	0.0190
	(0.0777)	(0.0605)	(0.0436)	(0.0382)	(0.0565)	(0.0606)	(0.0560)	(0.0558)
Inactive public river transportation (PRT) (0/1)	0.151*	-0.122	-0.147**	-0.0624	0.00821	-0.154**	-0.0909	0.0483
	(0.0897)	(0.0798)	(0.0588)	(0.0500)	(0.0749)	(0.0729)	(0.0813)	(0.0660)
Communication access (0/1)	-0.0167	-0.00787	-0.00434	-0.0111	-0.00908	0.00824	0.0820*	0.00920
	(0.0528)	(0.0452)	(0.0338)	(0.0256)	(0.0469)	(0.0421)	(0.0436)	(0.0346)
Indigenous (0/1)	-0.0543	0.0363	-0.0106	-0.00324	-0.0440	0.0318	0.121***	-0.0851**
	(0.0590)	(0.0451)	(0.0359)	(0.0332)	(0.0553)	(0.0523)	(0.0462)	(0.0427)
Population (log)	0.0718**	0.0258	-0.00289	0.0122	-0.0261	0.0250	-0.00440	0.00523
	(0.0330)	(0.0261)	(0.0177)	(0.0169)	(0.0300)	(0.0281)	(0.0282)	(0.0256)
Health facility (0/1)	0.0149	0.0244	0.0148	0.0368	0.0646	-0.0496	0.114	0.0151
	(0.0774)	(0.0633)	(0.0417)	(0.0361)	(0.0673)	(0.0670)	(0.0697)	(0.0599)
Secondary school (0/1)	0.0627	0.0284	0.0626	0.0176	0.0237	0.0397	0.0645	0.0791
	(0.0719)	(0.0532)	(0.0389)	(0.0324)	(0.0660)	(0.0634)	(0.0596)	(0.0488)
Church (0/1)	-0.0402	0.0672	0.0205	0.0148	-0.117*	0.139**	0.0850	0.0275
	(0.0761)	(0.0750)	(0.0568)	(0.0504)	(0.0636)	(0.0621)	(0.0605)	(0.0553)
River order 1 (0/1)	-0.122	0.0414	0.0437	0.0870	0.00260	0.0390	0.140*	-0.0364
	(0.0975)	(0.0764)	(0.0671)	(0.0601)	(0.0876)	(0.0860)	(0.0785)	(0.0669)
River order 2 (0/1)	-0.0287	0.0308	0.00206	0.0344	0.0334	-0.0303	0.0632	-0.0378
	(0.0714)	(0.0631)	(0.0570)	(0.0460)	(0.0710)	(0.0652)	(0.0664)	(0.0342)
Trade network with city market (0/1)	-0.0483	0.0423	0.0309	-0.000815	-0.000551	0.0330	0.0764	-0.00596
	(0.0600)	(0.0476)	(0.0402)	(0.0351)	(0.0545)	(0.0525)	(0.0493)	(0.0350)
Distance from district capital (log km)	0.0449	0.0162	-0.00840	-0.00663	0.00923	-0.0188	0.0146	0.0122
	(0.0290)	(0.0253)	(0.0180)	(0.0135)	(0.0301)	(0.0275)	(0.0264)	(0.0198)
Distance from nearest community (km)	-0.00699**	-0.00116	0.000437	0.0000778	0.000361	0.000130	0.00138	0.00193
	(0.00274)	(0.00316)	(0.00171)	(0.00138)	(0.00179)	(0.00196)	(0.00218)	(0.00245)
Community market (0/1)	-0.124	-0.0455	-0.00567	0.0209	-0.0243	0.0169	-0.0810	-0.00489
	(0.0909)	(0.0870)	(0.0627)	(0.0579)	(0.0637)	(0.0658)	(0.0687)	(0.0674)
Soccer network	0.00757	0.00189	-0.00198	-0.00416	0.00297	-0.00566	0.00432	0.00690
	(0.00985)	(0.00820)	(0.00606)	(0.00533)	(0.00889)	(0.00819)	(0.00789)	(0.00657)
Seed network (0/1)	-0.0169	-0.0280	-0.0335	-0.0373	0.0288	-0.0646	-0.00694	-0.0334
	(0.0450)	(0.0361)	(0.0279)	(0.0234)	(0.0426)	(0.0407)	(0.0375)	(0.0322)
Floodplain soil (0-1)	0.145	0.0469	0.0543	0.0327	0.200**	-0.145	-0.0262	0.0401
	(0.0942)	(0.0898)	(0.0655)	(0.0533)	(0.0886)	(0.0885)	(0.0797)	(0.0723)
Forest (0-1)	-0.119	-0.0461	0.125	-0.0202	0.264	-0.138	-0.218	-0.509**
	(0.221)	(0.149)	(0.125)	(0.113)	(0.220)	(0.217)	(0.174)	(0.203)
Basin fixed effects	YES	YES	YES	YES	YES	YES	YES	YES
Interviewer fixed effects	YES	YES	YES	YES	YES	YES	YES	YES
N	428	428	435	435	414	414	433	426
R squared	0.281	0.230	0.143	0.115	0.461	0.494	0.445	0.186
Mean of dependent variable	0.369	0.808	0.910	0.943	0.471	0.435	0.316	0.122
Sensitivity analysis: δ for $\beta=0$ given $Rmax$ ($=1.3R$):								
Distance from city (log km)	0.96	1.78	1.96		0.74		1.17	
Inactive PRT (0/1)	1.12		10.03			0.79		
Indigenous (0/1)							0.93	

Robust standard errors are shown in parentheses. *p<0.1, **p<0.05, ***p<0.01. The results for sensitivity analysis for predictors whose point estimates are statistically significant at least at a 10% significant level in Fig. 3 are reported (see Supplementary Note 2 for the sensitivity analysis).

Supplementary Table 2. OLS regression estimates for social behaviors

	Preventive measures before (z-score) (1)	Social restrictions at baseline (z-score) (2)	Preventive measures at follow-up (z-score) (3)	Social restrictions at follow-up (z-score) (4)
Distance from city (log km)	0.178**	0.161*	0.0945	0.104*
	(0.0854)	(0.0851)	(0.0590)	(0.0598)
Active public river transportation (0/1)	0.00507	0.0849	-0.00241	-0.0367
	(0.121)	(0.125)	(0.107)	(0.0836)
Inactive public river transportation (0/1)	0.118	-0.0320	-0.182	-0.113
	(0.166)	(0.157)	(0.114)	(0.116)
Communication access (0/1)	0.0999	0.178**	0.169***	0.0318
	(0.0872)	(0.0739)	(0.0641)	(0.0455)
Indigenous (0/1)	0.132	0.0255	0.0102	-0.106
	(0.101)	(0.0939)	(0.0774)	(0.0693)
Population (log)	0.0135	0.0181	0.00675	-0.0527*
	(0.0531)	(0.0525)	(0.0417)	(0.0310)
Health facility (0/1)	0.186	-0.115	-0.00431	-0.157*
	(0.127)	(0.136)	(0.0948)	(0.0899)
Secondary school (0/1)	-0.0633	-0.280**	-0.0588	-0.181**
	(0.106)	(0.109)	(0.0731)	(0.0799)
Church (0/1)	-0.0432	-0.264**	0.139	-0.183**
	(0.140)	(0.123)	(0.102)	(0.0883)
River order 1 (0/1)	-0.170	0.0795	-0.219*	0.0526
	(0.173)	(0.142)	(0.122)	(0.0964)
River order 2 (0/1)	-0.0145	0.0219	-0.0630	0.0796
	(0.119)	(0.107)	(0.0908)	(0.0615)
Trade network with city market (0/1)	-0.00217	-0.185**	-0.0691	0.0154
	(0.0888)	(0.0845)	(0.0709)	(0.0577)
Distance from district capital (log km)	-0.0260	0.0153	0.0157	0.00403
	(0.0481)	(0.0449)	(0.0323)	(0.0298)
Distance from nearest community (km)	0.00408	-0.00284	-0.00292	-0.00415
	(0.00403)	(0.00421)	(0.00312)	(0.00324)
Community market (0/1)	-0.0810	-0.0387	0.0200	0.145**
	(0.180)	(0.150)	(0.118)	(0.0701)
Soccer network	0.00515	0.0397***	0.0101	0.0161*
	(0.0167)	(0.0137)	(0.0134)	(0.00905)
Seed network (0/1)	0.00715	0.0182	0.0229	0.0285
	(0.0729)	(0.0670)	(0.0542)	(0.0453)
Floodplain soil (0-1)	-0.0764	0.0671	0.0867	0.0205
	(0.166)	(0.157)	(0.127)	(0.114)
Forest (0-1)	-0.164	-0.0752	-0.178	0.364
	(0.344)	(0.370)	(0.226)	(0.230)
Basin fixed effects	YES	YES	YES	YES
Interviewer fixed effects	YES	YES	YES	YES
N	408	417	434	431
R squared	0.572	0.603	0.682	0.813
Mean of dependent variable	0.169	-0.0645	-0.159	0.0624

Robust standard errors are shown in parentheses. *p<0.1, **p<0.05, ***p<0.01

Supplementary Table 3. OLS regression estimates for local private river transportation and government social assistance

	Local private river transportation				Government assistance		
	Any at baseline	Frequent at baseline	Any at follow-up	Frequent at follow-up	Cash by baseline	Food by baseline	Cash at follow-up
	(0/1)	(0/1)	(0/1)	(0/1)	(0/1)	(0/1)	(0/1)
	(1)	(2)	(3)	(4)	(5)	(6)	(7)
Distance from city (log km)	-0.0676	0.0335	0.0277	-0.0552	-0.0616	-0.0296	-0.0877***
	(0.0438)	(0.0446)	(0.0246)	(0.0465)	(0.0403)	(0.0479)	(0.0289)
Active public river transportation (PRT) (0/1)	0.133**	0.148**	0.137**	0.258***	0.00347	-0.200***	0.0183
	(0.0598)	(0.0700)	(0.0538)	(0.0620)	(0.0508)	(0.0581)	(0.0400)
Inactive public river transportation (PRT) (0/1)	-0.0811	-0.00351	-0.0308	0.170**	-0.449***	0.0317	-0.0480
	(0.0633)	(0.0783)	(0.0680)	(0.0778)	(0.0733)	(0.0750)	(0.0671)
Communication access (0/1)	-0.0237	-0.0853	-0.00902	-0.0830	0.0443	-0.0331	0.0622**
	(0.0345)	(0.0536)	(0.0174)	(0.0513)	(0.0440)	(0.0484)	(0.0273)
Indigenous (0/1)	-0.0470	-0.0423	-0.0246	0.00894	-0.0301	0.00644	0.0905***
	(0.0359)	(0.0529)	(0.0230)	(0.0526)	(0.0424)	(0.0490)	(0.0339)
Population (log)	-0.00181	0.0625*	0.0120	0.0464	-0.00767	0.0631**	0.0394*
	(0.0195)	(0.0329)	(0.0120)	(0.0298)	(0.0241)	(0.0273)	(0.0214)
Health facility (0/1)	0.0964*	0.0419	-0.0163	0.0138	0.0544	-0.00406	-0.0937**
	(0.0507)	(0.0750)	(0.0429)	(0.0656)	(0.0530)	(0.0682)	(0.0453)
Secondary school (0/1)	0.0155	0.00824	0.0142	0.0241	0.0194	-0.0993	-0.0230
	(0.0476)	(0.0692)	(0.0371)	(0.0621)	(0.0442)	(0.0684)	(0.0430)
Church (0/1)	0.0466	0.0764	-0.00426	0.0887	0.231***	-0.0635	0.0336
	(0.0522)	(0.0685)	(0.0425)	(0.0688)	(0.0663)	(0.0671)	(0.0411)
River order 1 (0/1)	0.121*	0.0734	0.00214	0.00210	-0.0370	0.212**	0.0134
	(0.0726)	(0.0869)	(0.0572)	(0.0823)	(0.0688)	(0.0859)	(0.0674)
River order 2 (0/1)	0.0491	0.0286	0.0237	0.118*	0.0627	0.0970	-0.0329
	(0.0500)	(0.0594)	(0.0363)	(0.0641)	(0.0561)	(0.0700)	(0.0340)
Trade network with city market (0/1)	-0.0236	0.0606	-0.0239	0.0788	-0.0171	0.0386	0.0261
	(0.0377)	(0.0526)	(0.0253)	(0.0571)	(0.0444)	(0.0564)	(0.0259)
Distance from district capital (log km)	-0.00424	-0.0377	0.00843	0.0563**	0.000103	0.0193	0.0387**
	(0.0181)	(0.0314)	(0.0122)	(0.0283)	(0.0209)	(0.0318)	(0.0175)
Distance from nearest community (km)	0.00149	0.00630***	-0.000308	-0.00530**	-0.000698	-0.00265	-0.000765
	(0.00189)	(0.00207)	(0.000907)	(0.00214)	(0.00261)	(0.00324)	(0.00114)
Community market (0/1)	0.000206	-0.0171	0.0460	0.0472	0.121*	-0.0194	0.0209
	(0.0688)	(0.0844)	(0.0441)	(0.0807)	(0.0713)	(0.0816)	(0.0463)
Soccer network	-0.00661	0.0124	-0.00266	-0.0101	0.00409	-0.00344	0.00235
	(0.00584)	(0.0102)	(0.00461)	(0.00955)	(0.00778)	(0.00937)	(0.00650)
Seed network (0/1)	-0.0106	0.0225	0.0117	0.0502	-0.00379	0.0242	0.0116
	(0.0281)	(0.0445)	(0.0210)	(0.0439)	(0.0324)	(0.0401)	(0.0257)
Floodplain soil (0-1)	-0.0290	-0.122	-0.00742	-0.139	0.129	0.0506	-0.180***
	(0.0693)	(0.0990)	(0.0540)	(0.0947)	(0.0893)	(0.0991)	(0.0646)
Forest (0-1)	0.0545	-0.169	0.0800	0.171	0.127	0.317	-0.0392
	(0.151)	(0.221)	(0.185)	(0.216)	(0.146)	(0.220)	(0.172)
Basin fixed effects	YES	YES	YES	YES	YES	YES	YES
Interviewer fixed effects	YES	YES	YES	YES	YES	YES	YES
N	429	429	427	427	422	423	433
R squared	0.382	0.250	0.315	0.355	0.364	0.154	0.368
Mean of dependent variable	0.860	0.333	0.930	0.541	0.818	0.780	0.118
Sensitivity analysis: δ for β=0 given *Rmax* (=*1.3R*):							
Distance from city (log km)							4.16
Active PRT (0/1)	0.97	1.34	1.09	6.51			
Inactive PRT (0/1)				97.15	1.28		
Indigenous (0/1)							16.67

Robust standard errors are shown in parentheses. *p<0.1, **p<0.05, ***p<0.01. The results for sensitivity analysis for predictors whose point estimates are statistically significant at least at a 10% significant level in Fig. 3 are reported (see Supplementary Note 2 for the sensitivity analysis).

Supplementary Table 4. OLS regression estimates for sample inclusion

	Baseline sample (0/1) (1)	Follow-up sample (0/1) (2)
Distance from city (log km)	-0.0304	-0.0537
	(0.0368)	(0.0370)
Public river transportation access (0/1)	-0.0132	-0.0136
	(0.0413)	(0.0416)
Telepone access (0/1)	0.0833**	0.0334
	(0.0344)	(0.0345)
Indigenous (0/1)	0.197***	0.176***
	(0.0363)	(0.0367)
Population	0.0313	0.0344*
	(0.0199)	(0.0199)
Health facility (0/1)	0.0358	0.0180
	(0.0552)	(0.0587)
Secondary school (0/1)	0.0908	0.111*
	(0.0559)	(0.0573)
River order 1 (0/1)	-0.119**	-0.126**
	(0.0603)	(0.0622)
River order 2 (0/1)	-0.0572	-0.0518
	(0.0420)	(0.0454)
Trade network with city market (0/1)	0.00633	-0.0268
	(0.0351)	(0.0365)
Distance from district capital (log km)	0.0313	0.0334*
	(0.0199)	(0.0202)
Distance from nearest community (log km)	-0.00288	-0.00239
	(0.00211)	(0.00201)
Community market (0/1)	0.0845*	0.0832*
	(0.0448)	(0.0456)
Soccer network	0.00657	0.00818
	(0.00708)	(0.00725)
Seed network (0/1)	0.0473	0.0633**
	(0.0300)	(0.0305)
Floodplain soil (0-1)	0.0553	0.0601
	(0.0636)	(0.0649)
Forest (0-1)	-0.245	-0.232
	(0.161)	(0.162)
Basin fixed effects	YES	YES
N	919	919
R squared	0.244	0.211
Mean of dependent variable	0.510	0.473

Robust standard errors are shown in parentheses. *p<0.1, **p<0.05, ***p<0.01

Supplementary Table 5. Definition and descriptive statistics of predictors

Variables	Definition	Mean	SD
Distance from city (log km)	River network distance from Iquitos or Pucallpa (closer one)	5.06	1.12
Communication acces (0/1)	Communication access (internet acces, cell phone acces, raidophone)	0.74	0.44
Internet acces (0/1)	Internet access (mostly cell phone access as well)[a]	0.14	0.34
Cell phone acces (0/1)	Cell phone access (but no internet access)[a]	0.32	0.47
Radiophone (0/1)	Radiophone	0.30	0.46
Indigenous (0/1)	Indigenous community (self-report)[b]	0.55	0.50
Population (log)	Population at baseline survey	5.16	0.96
Health facility (0/1)	Presence of health post	0.20	0.40
Secondary school (0/1)	Presence of secondary school	0.29	0.46
Church (0/1)	Presence of church	0.84	0.36
River order 1 (0/1)	River order 1	0.33	0.47
River order 2 (0/1)	River order 2	0.43	0.49
Trade network with city market (0/1)	Primary or secondary market is Iquitos or Pucallpa	0.63	0.48
Distance from district capital (log km)	River network distance from district capital	3.60	1.00
Distance from nearest community (log km)	River network distance from nearest community	5.59	6.85
Community market (0/1)	All five commodities (rice, sugar, cooking oil, soap, batteries) can be bought in community	0.93	0.26
Soccer network	Number of other communities with whom the community play soccer	6.03	2.53
Seed network (0/1)	Seeds and planting stock obtained from other communities or cities	0.48	0.50
Floodplain soil (0-1)	Land underlain by Holocene parent material (proportion in 5km buffer land)	0.62	0.30
Forest (0-1)	Land classified to be forest in 2015 (proportion in 5km buffer land)	0.82	0.17
No. communities		435	

[a] The number of observations are slightly smaller due to missing values. [b] One colonist community is treated as a mestizo community. See 'Empirical design' in Methods for the construction of some variables.

Supplementary Table 6. OLS regression estimates for public river transportation

	Available				Active at baseline				Active at follow-up			
	PRT	Large river boat	Small river boat	Speed boat	PRT	Large river boat	Small river boat	Speed boat	PRT	Large river boat	Small river boat	Speed boat
	(0/1)	(0/1)	(0/1)	(0/1)	(0/1)	(0/1)	(0/1)	(0/1)	(0/1)	(0/1)	(0/1)	(0/1)
	(1)	(2)	(3)	(4)	(5)	(6)	(7)	(8)	(9)	(10)	(11)	(12)
Distance to city (log km)	0.0360	0.133***	0.00866	0.0201	0.0496	0.0714*	0.0292	0.0371*	-0.0172	-0.00924	0.0397	0.0853**
	(0.0361)	(0.0354)	(0.0391)	(0.0545)	(0.0428)	(0.0388)	(0.0218)	(0.0223)	(0.0309)	(0.0319)	(0.0318)	(0.0334)
Communication access	0.0478	0.0504	0.000987	0.109**	0.0920***	0.0748**	-0.0133	0.00766	-0.00617	-0.0179	-0.0179	0.0633**
(0/1)	(0.0324)	(0.0359)	(0.0345)	(0.0522)	(0.0351)	(0.0372)	(0.00972)	(0.0239)	(0.0325)	(0.0305)	(0.0254)	(0.0317)
Indigenous (0/1)	0.00333	0.0215	-0.0282	0.0101	-0.0311	0.00287	-0.0454*	-0.0673**	0.0204	0.0686*	0.0355	-0.0731*
	(0.0413)	(0.0448)	(0.0408)	(0.0618)	(0.0408)	(0.0358)	(0.0263)	(0.0341)	(0.0349)	(0.0377)	(0.0263)	(0.0389)
Population (log)	0.0464**	0.0604***	0.0459**	-0.0663**	-0.0450*	-0.0438**	0.00547	-0.0157	0.0312	0.0245	-0.0154	-0.00876
	(0.0214)	(0.0226)	(0.0217)	(0.0298)	(0.0236)	(0.0219)	(0.0117)	(0.0144)	(0.0193)	(0.0173)	(0.0139)	(0.0172)
Health facility (0/1)	0.0759	0.0489	0.0126	0.0966	0.00284	-0.00977	0.00384	0.0202	0.0502	0.0731	-0.00157	0.0937**
	(0.0579)	(0.0636)	(0.0508)	(0.0750)	(0.0507)	(0.0507)	(0.0272)	(0.0438)	(0.0486)	(0.0487)	(0.0319)	(0.0455)
Secondary school (0/1)	-0.0264	-0.0716	-0.0231	0.0466	-0.000764	-0.0451	0.00224	0.0436	0.00603	0.0177	0.0281	0.00777
	(0.0453)	(0.0505)	(0.0501)	(0.0661)	(0.0420)	(0.0431)	(0.0284)	(0.0362)	(0.0461)	(0.0465)	(0.0258)	(0.0387)
Church (0/1)	0.144**	0.101*	-0.00385	0.123*	0.0738	0.00547	-0.0138	0.0190	0.0246	-0.0475	0.0294	-0.0117
	(0.0610)	(0.0602)	(0.0556)	(0.0704)	(0.0474)	(0.0499)	(0.0279)	(0.0389)	(0.0502)	(0.0517)	(0.0275)	(0.0449)
River order 1 (0/1)	0.159**	0.267***	0.188***	0.173*	-0.101	-0.0570	0.0159	0.00352	-0.0615	-0.0529	-0.0312	-0.0181
	(0.0723)	(0.0815)	(0.0688)	(0.101)	(0.0700)	(0.0632)	(0.0403)	(0.0402)	(0.0595)	(0.0660)	(0.0452)	(0.0529)
River order 2 (0/1)	0.00814	0.124**	0.0487	0.0680	-0.197***	-0.0761	-0.0362	-0.0598**	-0.0670	0.00948	-0.0276	-0.0453
	(0.0518)	(0.0584)	(0.0471)	(0.0810)	(0.0577)	(0.0513)	(0.0252)	(0.0232)	(0.0443)	(0.0418)	(0.0365)	(0.0374)
Trade network with city	0.0302	0.0310	0.0362	0.0710	-0.00659	0.0235	0.00734	-0.0225	-0.0559	-0.0390	0.0115	-0.0378
market (0/1)	(0.0398)	(0.0389)	(0.0412)	(0.0604)	(0.0386)	(0.0392)	(0.0217)	(0.0260)	(0.0345)	(0.0264)	(0.0293)	(0.0341)
Distance from district	-0.0276	-0.00223	-0.0243	-0.0150	0.0274	0.0278	0.00296	-0.00723	-0.00667	-0.0188	-0.00902	-0.0101
capital (log km)	(0.0212)	(0.0232)	(0.0167)	(0.0308)	(0.0190)	(0.0200)	(0.00857)	(0.0147)	(0.0195)	(0.0192)	(0.0115)	(0.0158)
Distance from nearest	0.00438**	0.00399*	0.00223	-0.00210	-0.000618	-0.000173	0.000917	0.00175*	0.00159	0.000118	0.000777	0.00349*
community (km)	(0.00196)	(0.00218)	(0.00209)	(0.00370)	(0.00224)	(0.00205)	(0.000706)	(0.000973)	(0.00265)	(0.00161)	(0.000942)	(0.00180)

(continued)

	Available				Active at baseline				Active at follow-up			
	PRT	Large river boat	Small river boat	Speed boat	PRT	Large river boat	Small river boat	Speed boat	PRT	Large river boat	Small river boat	Speed boat
	(0/1)	(0/1)	(0/1)	(0/1)	(0/1)	(0/1)	(0/1)	(0/1)	(0/1)	(0/1)	(0/1)	(0/1)
	(1)	(2)	(3)	(4)	(5)	(6)	(7)	(8)	(9)	(10)	(11)	(12)
Community market (0/1)	0.204***	0.129**	0.0672	0.0617	0.106**	-0.0489	0.00249	-0.0175	-0.0183	-0.0648	0.0253	0.0183
	(0.0540)	(0.0597)	(0.0676)	(0.0707)	(0.0449)	(0.0461)	(0.0311)	(0.0327)	(0.0478)	(0.0408)	(0.0386)	(0.0349)
Soccer network	0.000115	-0.00452	0.00840	0.0153	0.0214***	0.0242***	0.00640**	0.00601	0.00148	-0.00325	0.00248	-0.00180
	(0.00647)	(0.00718)	(0.00695)	(0.0101)	(0.00711)	(0.00702)	(0.00285)	(0.00410)	(0.00662)	(0.00592)	(0.00390)	(0.00580)
Seed network (0/1)	-0.0373	-0.0378	-0.0119	-0.0716	-0.0757**	-0.0662**	-0.000424	0.0204	-0.00316	0.0256	-0.00485	-0.0176
	(0.0314)	(0.0343)	(0.0326)	(0.0455)	(0.0311)	(0.0310)	(0.0173)	(0.0207)	(0.0281)	(0.0261)	(0.0225)	(0.0268)
Floodplain soil (0-1)	0.203**	0.274***	0.0486	0.0207	0.166**	0.269***	-0.0365	-0.00492	0.117*	0.130*	-0.0884**	-0.0188
	(0.0841)	(0.0876)	(0.0742)	(0.105)	(0.0745)	(0.0756)	(0.0312)	(0.0404)	(0.0634)	(0.0699)	(0.0399)	(0.0557)
Forest (0-1)	0.508***	0.134	0.611***	0.349	0.0145	-0.0878	0.0827	-0.106	0.0613	-0.0471	-0.172	-0.291**
	(0.183)	(0.167)	(0.184)	(0.234)	(0.159)	(0.154)	(0.0870)	(0.112)	(0.126)	(0.139)	(0.105)	(0.128)
Available large river boat (0/1)					0.326***	0.665***	0.0570*	0.00146	0.323***	0.666***	0.0264	0.0252
					(0.0536)	(0.0484)	(0.0333)	(0.0366)	(0.0524)	(0.0527)	(0.0236)	(0.0419)
Available small river boat (0/1)					0.235***	-0.121**	0.849***	-0.0305	0.511***	0.107***	0.842***	-0.00408
					(0.0447)	(0.0503)	(0.0407)	(0.0282)	(0.0523)	(0.0404)	(0.0445)	(0.0505)
Available speed boat (0/1)					0.394***	0.299***	-0.0194	0.864***	0.0924***	0.0177	-0.0484**	0.730***
					(0.0408)	(0.0383)	(0.0182)	(0.0273)	(0.0354)	(0.0338)	(0.0244)	(0.0363)
Basin fixed effects	YES	YES	YES	YES	YES	YES	YES	YES	YES	YES	YES	YES
Interviewer fixed effects	YES	YES	YES	YES	YES	YES	YES	YES	YES	YES	YES	YES
N	435	435	435	435	435	435	435	435	435	435	435	435
R squared	0.461	0.535	0.545	0.253	0.650	0.678	0.886	0.849	0.693	0.745	0.804	0.712
Mean of dependent variable	0.798	0.678	0.285	0.414	0.641	0.492	0.253	0.366	0.669	0.515	0.257	0.324

The estimated predictors of access to public river transportation (PRT) and active PRT at the baseline and follow-up surveys are shown. Robust standard errors are shown in parentheses. *p<0.1, **p<0.05, ***p<0.01

Supplementary Table 7. OLS regression estimates for return migration

	Return migration					
	Any	To reduce infection risk	To get help from people in communities	To secure food	To secure livelihoods	To maintain social life
	(0/1)	(0/1)	(0/1)	(0/1)	(0/1)	(0/1)
	(1)	(2)	(3)	(4)	(5)	(6)
Distance from city (log km)	-0.00418	0.0434	0.00463	0.00514	0.00295	0.0312
	(0.0327)	(0.0353)	(0.0263)	(0.0320)	(0.0347)	(0.0242)
Active public river transportation (0/1)	0.0875	0.122**	0.0912*	0.0601	0.0699	-0.000197
	(0.0532)	(0.0612)	(0.0542)	(0.0477)	(0.0617)	(0.0415)
Inactive public river transportation (0/1)	0.0241	-0.0500	0.0305	-0.00119	0.00511	-0.0122
	(0.0668)	(0.0829)	(0.0652)	(0.0760)	(0.0776)	(0.0636)
Communication access (0/1)	-0.0376	0.00860	0.0345	-0.0173	-0.0252	-0.0552**
	(0.0254)	(0.0281)	(0.0314)	(0.0261)	(0.0328)	(0.0243)
Indigenous (0/1)	0.0251	-0.0641*	0.0464	0.0453	0.0313	0.0106
	(0.0292)	(0.0368)	(0.0367)	(0.0352)	(0.0364)	(0.0325)
Population (log)	0.0267	0.0390*	0.000986	0.0233	0.0164	0.00199
	(0.0220)	(0.0220)	(0.0233)	(0.0229)	(0.0234)	(0.0165)
Health facility (0/1)	0.0380	-0.0473	-0.114**	0.0569	0.00759	0.0896**
	(0.0527)	(0.0566)	(0.0572)	(0.0518)	(0.0627)	(0.0411)
Secondary school (0/1)	-0.0355	-0.0397	0.0275	-0.0573	-0.0303	0.0179
	(0.0509)	(0.0485)	(0.0548)	(0.0452)	(0.0558)	(0.0469)
Church (0/1)	-0.0343	-0.0253	0.0165	-0.0133	-0.00604	0.0340
	(0.0511)	(0.0513)	(0.0601)	(0.0470)	(0.0553)	(0.0394)
River order 1 (0/1)	-0.0136	0.0590	0.0388	-0.0568	0.0307	-0.109
	(0.0631)	(0.0751)	(0.0658)	(0.0635)	(0.0746)	(0.0675)
River order 2 (0/1)	0.0813**	0.0524	-0.00668	0.0597	0.0967**	-0.0164
	(0.0398)	(0.0457)	(0.0334)	(0.0386)	(0.0420)	(0.0375)
Trade network with city market (0/1)	0.0320	0.0154	0.0700*	0.0502	0.0451	0.0448
	(0.0335)	(0.0375)	(0.0386)	(0.0314)	(0.0401)	(0.0300)
Distance from district capital (log km)	0.0132	0.0218	0.0234	-0.00703	0.00902	-0.0386**
	(0.0130)	(0.0144)	(0.0167)	(0.0117)	(0.0175)	(0.0158)
Distance from nearest community (km)	-0.00106	-0.000974	-0.00271*	0.000473	0.00146	-0.00141
	(0.00151)	(0.00143)	(0.00152)	(0.00107)	(0.00176)	(0.00115)
Community market (0/1)	-0.0504	0.0761	-0.0127	-0.0288	-0.0193	-0.0362
	(0.0462)	(0.0666)	(0.0591)	(0.0396)	(0.0646)	(0.0457)
Soccer network	-0.00709	-0.00805	-0.00427	-0.00446	-0.00677	-0.00831
	(0.00545)	(0.00660)	(0.00729)	(0.00637)	(0.00665)	(0.00583)
Seed network (0/1)	-0.0374	-0.0223	-0.0202	0.00465	-0.0386	-0.0186
	(0.0260)	(0.0292)	(0.0284)	(0.0269)	(0.0297)	(0.0256)
Floodplain soil (0-1)	0.0342	0.0488	-0.106	0.0607	0.0549	0.0711
	(0.0622)	(0.0793)	(0.0675)	(0.0547)	(0.0660)	(0.0651)
Forest (0-1)	-0.00176	-0.360**	-0.0501	0.0397	-0.0691	0.185
	(0.150)	(0.166)	(0.149)	(0.163)	(0.164)	(0.173)
Basin fixed effects	YES	YES	YES	YES	YES	YES
Interviewer fixed effects	YES	YES	YES	YES	YES	YES
N	434	434	434	434	434	434
R squared	0.743	0.553	0.620	0.724	0.640	0.624
Mean of dependent variable	0.449	0.237	0.293	0.341	0.406	0.191

Robust standard errors are shown in parentheses. *p<0.1, **p<0.05, ***p<0.01
